# Bovistol B, bovistol D and strossmayerin: Sesquiterpene metabolites from the culture filtrate of the basidiomycete *Coprinopsis strossmayeri*

**DOI:** 10.1371/journal.pone.0229925

**Published:** 2020-04-06

**Authors:** Alice M. Banks, Lijiang Song, Gregory L. Challis, Andy M. Bailey, Gary D. Foster

**Affiliations:** 1 School of Biological Sciences, University of Bristol, Bristol, United Kingdom; 2 Department of Chemistry, University of Warwick, Coventry, United Kingdom; Ruhr-Universitat Bochum, GERMANY

## Abstract

Basidiomycete fungi are a rich source of natural products with a diverse array of potentially exploitable bioactivities. Two dimeric sesquiterpenes, bovistol B (**1**) and D (**2**), and one monomeric sesquiterpene, strossmayerin (**7**), were isolated from the culture filtrate of the basidiomycete fungus *Coprinopsis strossmayeri*. The structures were determined through a combination of MS and 1D/2D NMR spectroscopic techniques. Likely monomeric precursors, identified on the basis of HRMS analysis, allow a plausible biosynthetic pathway to be proposed for the biosynthesis of **1** and **2**, involving the dimerisation of the monomer through a hetero-Diels-Alder mechanism. A gene cluster, including a putative sesquiterpene 1–11 cyclase, was identified through phylogenetic and RNA-seq analysis, and is proposed to be responsible for the biosynthesis of **1** and **2**.

## Introduction

The basidiomycete fungi are a phylum of organisms with a hugely diverse range of biological capabilities [1]. Although the secondary metabolism potential of basidiomycetes has long been known, inherent difficulties associated with the cultivation of such species, for example limited genetic tractability [2], have hindered extensive investigation. In recent years, some of these practical constraints have been overcome [3], making it timely to reconsider this understudied phylum.

We have recently re-evaluated the coprinoid basidiomycete *Coprinopsis strossmayeri* [4], previously *Coprinus quadrifidus* [5]; a species in which antimicrobial activity had been briefly reported, though not thoroughly investigated [6]. The coprinoid fungi encompass the *Coprinopsis*, *Coprinus*, *Coprinellus*, and *Parasola* genera in the Psathyrellaceae family. Generally favouring a habitat in herbivore dung, these organisms have evolved to live in highly competitive environments. Notable bioactive metabolites previously reported from coprinoid species include: the antimicrobial sesquiterpene illudins, illudin I, I2, J and J2, from *Coprinopsis episcopalis* [7,8]; the diterpene heptemerones A-G from *Coprinus heptemerus* [9], as well as the sesquiterpene antibiotic coprinol from fermentations of *Coprinus* sp. [10]. Basidiomycetes are prolific producers of terpenoid compounds, in particular the sesquiterpenes [11]–a group of highly bioactive compounds with a wide range of activities and applications ranging from pharmaceutical to agricultural [12].

In our ongoing search for novel antimicrobials, investigations of the culture filtrate of *C*. *strossmayeri* were carried out. This led to the isolation of two novel compounds, **2** and **7**, as well as the identification of **1** previously isolated from *Cyclocybe aegerita* [13], the structures of which were determined based on spectroscopic data. Compounds were screened for antimicrobial activity against *Bacillus subtilis* ATCC 6633, *Escherichia coli* DH5α and *Saccharomyces cerevisiae* Y10000. RNA-seq data led to the identification of a proposed gene cluster responsible for the biosynthesis of **1** and **2**.

## Materials and methods

### Producing organism

Isolate CBS 177.39, listed as *Coprinus quadrifidus*, was obtained from The CBS Fungal Biodiversity Centre and its internal transcribed spacer region (S1 Text in S1 File) confirmed the isolate as *Coprinopsis strossmayeri*, showing 99.69% identity to *C*. *strossmayeri* isolate PK7630 (accession number MH752456.1). The fungus was maintained on potato dextrose agar (potato dextrose broth 24 g/L, agar 15 g/L) at 20°C.

### Fermentation and isolation

A 1 L culture of *C*. *strossmayeri* was grown in potato dextrose broth (PDB) (potato dextrose broth 24 g/L) for 14 days at 20°C at 170 rpm. Fungal material was removed *via* filtration through Miracloth, culture filtrate was acidified to pH 3.0 with HCl, EtOAc was added to the culture filtrate at a 1:1 (v:v) ratio and mixed for 30 minutes then vacuum filtered to remove remaining mycelial fragments. The filtrate was separated using a separation funnel and the organic phase collected. The aqueous phase was extracted two further times and the extracts combined. Water was removed from the pooled solvent fraction using anhydrous MgSO_4_ before drying using a rotary evaporator. This yielded 400 mg total crude extract.

A 90 mg sample of crude extract was initially assessed and fractionated using preparative HPLC. A Waters 2767 Sample Manager with Waters 2545 pump system, Phenomenex LUNA column (5 μm, C_18_, 100 Å, 10 × 250 mm) was used for reverse-phase chromatography, with Phenomenex Security Guard precolumn (Luna C_5_ 300 Å). UV absorbance was detected between 200–400 nm with Waters 2998 diode array detector; mass spectrometry with Waters Quattro Micro; and approximate target compound abundance evaluated with Waters 2424 for ELSD. Mobile phases consisted of A: water with 0.05% formic acid; and B: acetonitrile with 0.05% formic acid. A gradient of 20% B to 90% B in 30 minutes was employed with flow rate of 5 mL/min. Compounds from the five major peaks were collected across five fractions (A-E) giving the following yields: A = 1.6 mg, B = 0.5 mg, C = 1.3 mg, D = 2.8 mg, E = 3 mg.

LC-MS analysis was performed on the fractions with Dionex 3000RS UHPLC coupled with Bruker MaXis Impact Q-TOF mass spectrometer. An Agilent Zorbax Eclipse plus column (C18, 100 x 2.1 mm, 1.8 μm) was used. Mobile phases consisted of A: water with 0.1% formic acid; and B: acetonitrile with 0.1% formic acid. After 5 minutes of isocratic run at 5% B, a gradient of 5% B to 100% B in 15 minutes was employed with flow rate at 0.2 mL/min, UV was set at 210 nm. Mass spectrometer was operated in electrospray positive ion mode with a scan range 50–3,000 *m/z*. Source conditions ar:, end plate offset at -500 V; capillary at -4,500 V; nebulizer gas (N_2_) at 1.4 bar; dry gas (N_2_) at 8 L/min; dry temperature at 180°C. Ion transfer conditions as, ion funnel 1 RF at 200 Vpp; ion funnel 2 RF at 200 Vpp, hexapole RF at 200 Vpp; quadruple ion energy at 5 ev, quadrupole low mass set at 55 *m/z*; collision energy at 5.0 ev; collision RF ramping from 800 to 1500 Vpp; transfer time set at from 100 to 155 μs; pre-Pulse storage time set at 5 μs. Calibration was done with sodium formate (10 mM) through a loop injection of 20 μL of standard solution at the beginning of each run.

Each compound was further purified using an Agilent Zorbax C18 column (100 x 21.1 mm, 5 μm) connected to an Agilent 1100 HPLC at a flow rate of 5 mL/min, monitoring absorbance at 210 nm. Mobile phases consisted of A: water containing 0.1% formic acid; and B: acetonitrile containing 0.1% formic acid. The following program was used to elute the column: 0 min, 80% B; 5 min, 80% B; 25 min, 100% B; 30 min, 100% B; 33 min 80% B; 38 min 80% B. Fractions containing target compounds were identified using ESI-HR-Q-TOF-MS and pooled. Organic solvent was removed using a rotary evaporator and the resulting aqueous solutions freeze dried yielding 2 mg each of **1** and **2** and 1 mg of **7**. Samples were analysed immediately by NMR spectroscopy.

### Compound identification

^1^ H, COSY, HSQC and HMBC NMR spectra were acquired in d_4_- MeOH (180 μL in 3 mm tube) on a Bruker Avance II 700 MHz spectrometer equipped with a TCI cryoprobe at 298 K. The solvent peak was used to calibrate the spectra.

### Antimicrobial screening

Chemical fractions obtained by preparative HPLC were dissolved to a concentration of 1 mg/mL in dimethyl sulfoxide. Microbial plates were prepared in 90 mm Petri dishes by resuspending microbial cells in the appropriate growth medium supplemented with 2, 3, 5-triphenyl-2H-tetrazolium chloride (200 μg/mL)–*B*. *subtilis* ATCC 6633 (tryptic soy broth 30 g/L, agar 5 g/L); *E*. *coli* DH5α (LB Broth, Miller 25 g/L, agar 5 g/L); *S*. *cerevisiae* Y10000 (yeast extract 10 g/L, peptone 20 g/L, D-glucose 20 g/L, adenine hemisulphate 40 mg/L, agar 5 g/L). 50 μl of each fraction was aliquoted into centrally bored wells in each assay plate and the plates incubated appropriately (*B*. *subtilis* and *S*. *cerevisiae* 48 hours at 28°C, *E*. *coli* 24 hours at 37°C). Antimicrobial activity was determined by the presence of a zone of inhibition surrounding the central well.

### Transcriptome analysis

RNA was extracted from *C*. *strossmayeri* mycelium, harvested from a two-week culture grown in PDB, using the E.Z.N.A^®^ Fungal RNA Kit (OMEGA bio-tek). Isolated RNA was quality checked using RNA Analysis ScreenTape^®^ (Agilent). Approximately 500 ng of total RNA was prepared for sequencing using the Illumina TruSeq Total RNA LT Kit (Illumina). The data were processed using RTA version 1.18.64, with default filter and quality settings. The reads were demultiplexed with CASAVA 1.8.4, allowing no mismatches. This was carried out at the Bristol Genomics Facility. RNA-seq data were processed using Galaxy QC and manipulation tools to trim the sequences followed by the TopHat RNA analysis tool to map the RNA-seq reads to the assembled genomes. These data were then viewed in Artemis to evaluate relevant expression levels of genes of interest. Partek^®^ Genomics Suite was also used to map RNA-seq reads to assembled genomes and to genes of interest. RNA-seq data are available on the NCBI SRA database under the accessions: STUDY: PRJNA604530; SAMPLE: CBS 177.39 (SAMN13973684); EXPERIMENT: C.s (SRX7684385); RUN: AB_C_ACAGTG_L001_R1_001.fastq (SRR11032120).

## Results and discussion

Crude ethyl acetate extract from the culture filtrate of *C*. *strossmayeri* was fractionated using preparative HPLC. Five fractions were obtained, A-E, each containing one of the major peaks. These were examined for inhibitory activity against *B*. *subtilis* ATCC 6633, *E*. *coli* DH5α and *S*. *cerevisiae* Y10000 using plate-based bioassays. Weak antimicrobial activity was detected in fractions A and E against *B*. *subtilis* (S20 Fig in S1 File) but not *E*. *coli* or *S*. *cerevisiae*. No antimicrobial activity was observed in fractions B-D.

Initial preparative HPLC fractions were analysed using LC-MS and major components were further purified with semi-preparative HPLC. Structural elucidation was carried out with a combination of HRMS and 1D/2D NMR spectroscopic techniques; this led to the identification of **1** from fraction E and two novel compounds, **2** and **7**, from fractions D and C respectively. Fraction E was identified as the dimeric sesquiterpene bovistol B (**1**) and fraction D the related bovistol D (**2**), both yellowish-white solids. Fraction C was identified as a sesquiterpene monomer strossmayerin (**7**). Compounds present in fractions A and B were produced at too low titre to yield sufficient product for structural elucidation.

Bovistol B: High resolution MS data established **1** with the molecular formula C_30_H_38_O_5_, (measured *m/z* 479.2793, calculated (C_30_H_38_O_5_+H)^+^ 479.2792) with 12 degrees of unsaturation. The UV λmax at 290 nm also indicates the presence of an aromatic ring or conjugated multiple double bonds. ^1^ H, ^13^ C, COSY, HSQC and HMBC NMR spectra were acquired in d4- MeOH (S2-S5 Figs in S1 File). There are 2 methyl groups appearing as a singlet (1.14 and 1.16 ppm) connected to saturated quaternary carbons, while the third methyl group (2.13 ppm), also appears as a singlet, is probably connected to an aromatic ring or substituted carbon-carbon double bond. There are two isolated oxygenated methylene signals H10 (3.40 ppm) and H10’ (3.41 ppm) and five further isolated methylene signals (H6, H8, H6’, H8’ and H12’) with no COSY correlations observed to any other signals. COSY correlations established the connectivity between H14 (3.50 ppm) and H13 (2.80 ppm), H14’ (0.80/1.13 ppm) and H13’ (0.26/1.38 ppm) and H15 (2.77 ppm) with H15’ (1.87/2.19 ppm). HMBC correlations observed from H10 and H11 to C6, C7 and C8 confirmed the presence of a five membered ring. Further HMBC correlation from H12 to C3, C5, from H8 to C1, C9 and from H13 to C2, C4 and C14 established coupling of the aromatic ring with the 5-membered ring and complete the right-hand part of the structure. The left-hand part of the structure was also deduced mainly from HMBC correlations, similar correlations confirmed the presence of a 5-membered ring. Different to the right part of the structure, there is an isolated olefinic CH_2_ signal (5.25ppm with carbon chemical shift at 113.3 ppm) instead of a methyl group connected to C4’, HMBC correlation from H12’ to C3’ and C5’confirmed the presence of the isolated double bond. The relatively high field methylene signal at 0.26/1.38 ppm (H13’) and 0.80/1.13 ppm (H14’) suggest the presence of a cyclopropane group. HMBC correlation from H13’ and H14’ to C4’, C3’ and C2’ established the left-hand side of the structure. Key HMBC correlation observed from H15’ to C1’, C2’ and C3’ from the left side and C15, C2 from right-hand side established the connectivity between the two monomers.

**Fig 1:**
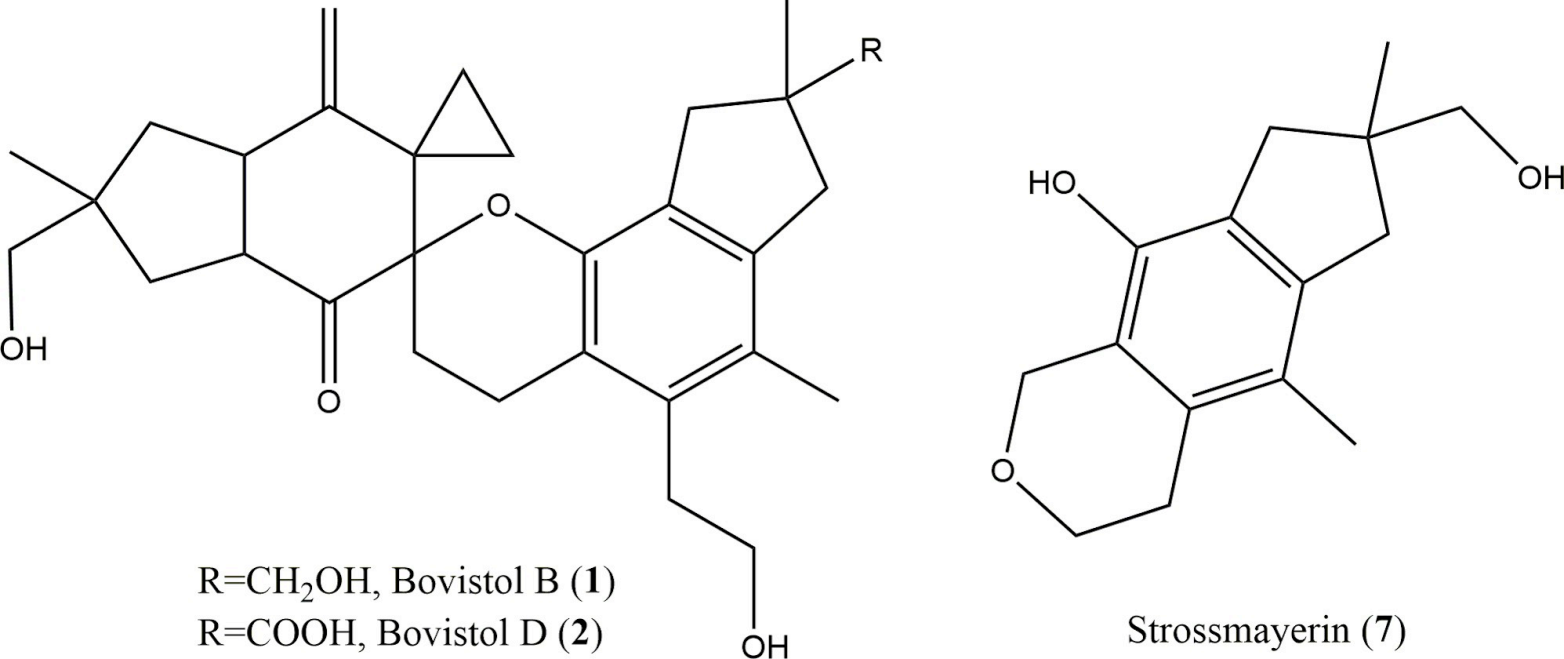
Novel sesquiterpenes produced by *C*. *strossmayeri*. Structures of bovistol B (**1**), D (**2**) and strossmayerin (**7**).

Therefore, the planar structure of **1** is proposed as a dimeric sesquiterpene; the structure of **2** was elucidated as the oxidised form of **1**, where the C10 hydroxymethyl group is oxidised to a carboxyl group. **1**, previously isolated from *C*. *aegerita*, has been characterised by MS and NMR spectroscopy and the relative configurations assigned by ROSEY correlations [13]. The closely related compound bovistol (**8**) has also undergone extensive structural characterisation [14]. **7** showed high similarity with the right-hand side of compound **1** and appears to be derived from delta 6-protoilludene **3** (Fig 1; S1, S7 and S13 Figs in S1 File).

**Fig 2:**
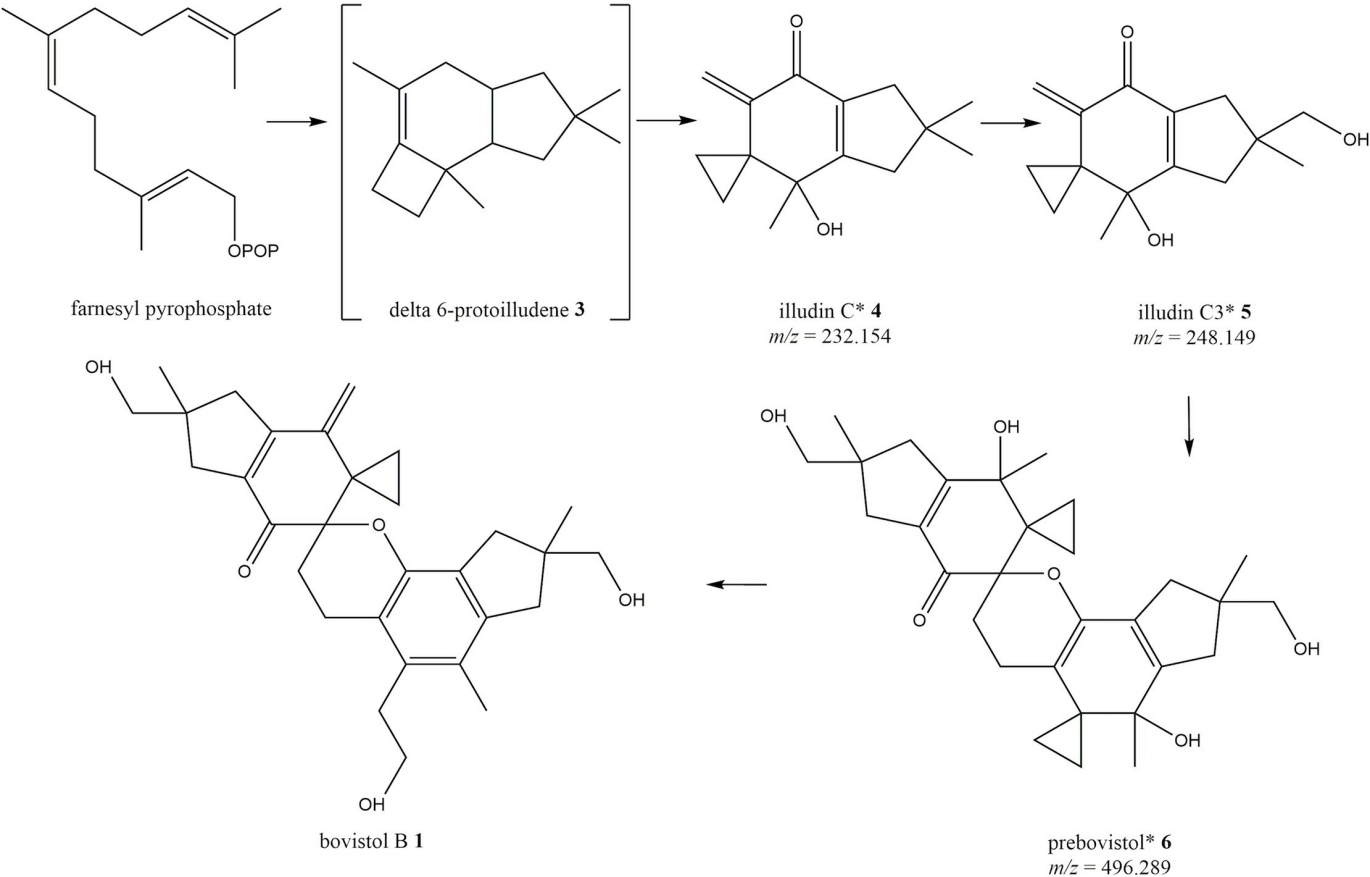
Proposed biosynthetic pathway for bovistol B (1). Accurate masses and formulae corresponding to the compounds indicated with an asterisk were identified in the HRMS data.

**1** and **2** appear to be dimeric sesquiterpenes, a proposed pathway for their biosynthesis is presented in Fig 2. Pathway intermediates to support this, identified from HRMS data, are indicated with asterisks in Fig 2. The proposed biosynthesis starts with a 1,11 cyclisation of farnesyl diphosphate to give delta 6-protoilludene (**3**), this is then oxidised to illudin C (**4**), then illudin C3 (**5**). A dimerisation then takes place to yield prebovistol (**6**), this asymmetric dimerisation is predicted to proceed via an inverse-electron demand hetero-Diels-Alder (DA) mechanism.

**Fig 3:**
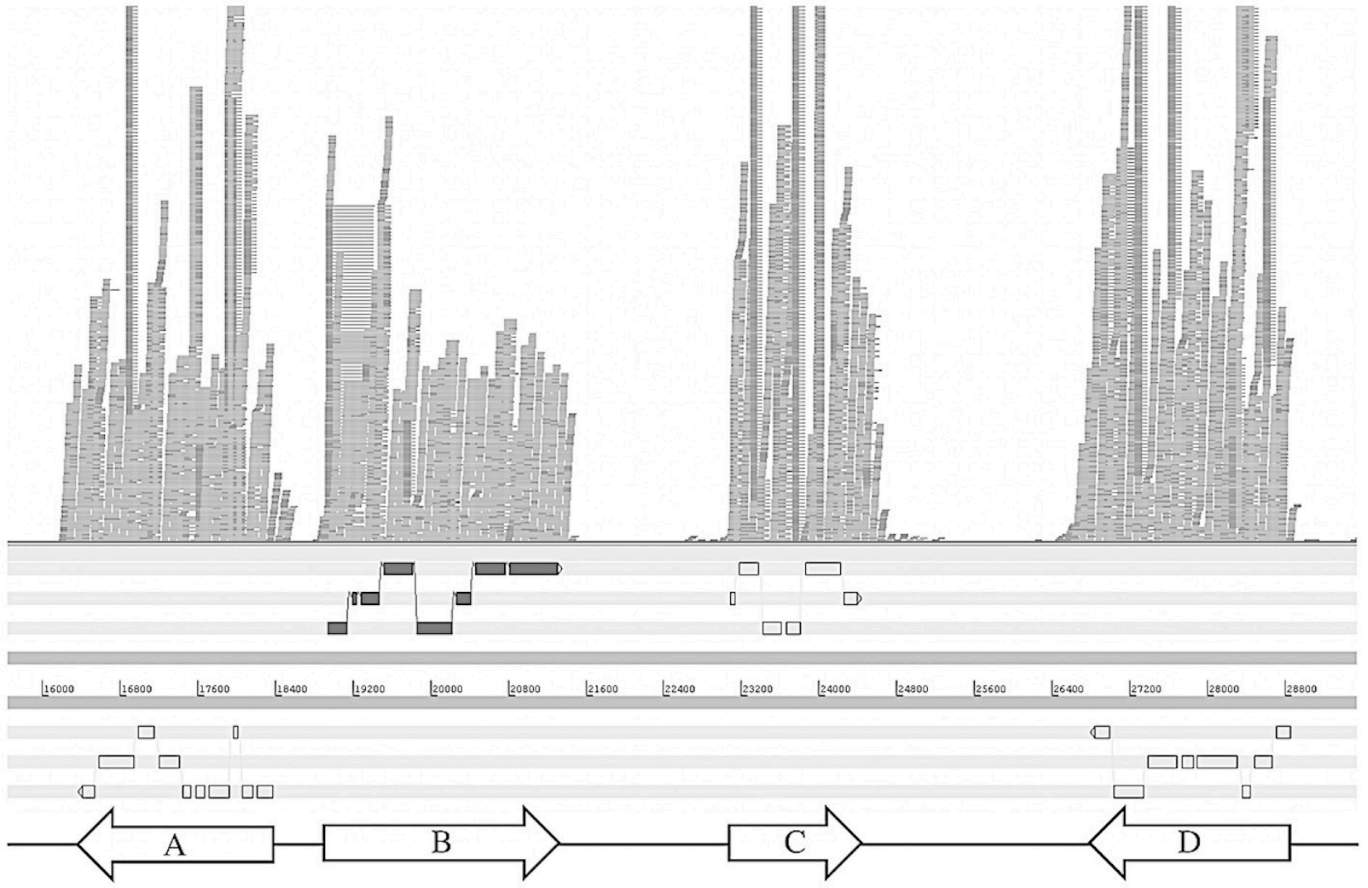
Putative gene cluster for the biosynthesis of 1. RNA-seq data aligned to the *C*. *strossmayeri* genome to identify cDNA sequences of the genes comprising the putative gene cluster for the biosynthesis of **1**. Genes present include (A) FAD oxidoreductase, (B) sesquiterpene cyclase, (C) aldo-keto reductase, (D) P450-dependent oxidoreductase.

Phylogenetic analysis has shown basidiomycete sesquiterpene cyclases to form distinct clades corresponding to the mode of cyclisation [15,16]. The *C*. *strossmayeri* genome [5] was mined for sesquiterpene biosynthesis genes using antiSMASH [17–19], revealing four sesquiterpene synthases. These genes were compared to other characterised basidiomycete sesquiterpene synthases [15,16], using neighbor-joining in MEGA 5.0 [20,21]. Protein sequences are available in S2 Text in S1 File. This revealed only one likely gene capable of carrying out the 1,11 cyclisation required (S19 Fig), this was located in contig 98 (FTPT010000098) of the genome assembly [5]. Analysis of genes adjacent to this sesquiterpene cyclase, along with RNA-seq analysis performed through Galaxy and Artemis [22–24], led to the determination of a candidate gene cluster responsible for the biosynthesis of **1** (Fig 3), along with the proposed cDNA sequences of each gene. This cluster comprises a core sesquiterpene cyclase (FTPT010000098 bp 18,942–22,551), FAD oxidoreductase (FTPT010000098 bp 14,417–18,361), aldo-keto reductase (FTPT010000098 bp 23,093–24,388), and cytochrome P450-dependent oxidoreductase (FTPT010000098 bp 26,838–28,847). Genomic DNA sequences are available in S3 Text in S1 File. If the DA reaction is enzyme mediated, the gene responsible was not immediately apparent. Few such enzymes have been characterised to date [25–27], so motifs responsible for such activity are yet to be defined.

We are aware that this is not the first report of dimeric sesquiterpenes from basidiomycete fungi (e.g. spirodienone) [28], however, to our knowledge this is the first where an inverse-electron demand hetero-DA mechanism has been proposed for the dimerisation. It is noteworthy that **5** has been reported by other authors without any observation of dimerisation, so it is feasible that the dimerisation is enzyme-mediated and if so, such mechanisms would certainly be of interest given the scarcity of DA enzymes reported to date. The pericyclase IccD from the ascomycete *Penicillium variabile* catalyses the inverse-electron demand DA reaction in the biosynthesis of ilicicolin [29], however, no significant homology to IccD was found in the *C*. *strossmayeri* genome flanking the putative gene cluster for the biosynthesis of **1**. The S-adenosyl-L-methionine (SAM)-dependent enzyme, LepI from *Aspergillus nidulans*, can catalyse intramolecular DA and hetero-DA reactions involved in the biosynthesis of leporin B [30], but similarly, no significant homology was found with genes present in the *C*. *strossmayeri* genome when a tblastn search was performed. There are increasing reports of characterised intramolecular Diels-Alderases of natural origin [31], however many originate from bacterial or ascomycete hosts and are likely to differ significantly to those responsible for pericyclic activity in basidiomycete natural product biosynthesis. This work highlights the vastly understudied basidiomycetes as a promising source of novel antimicrobial compounds.

## Supporting information

S1 File (PDF)

## References

[1] de Mattos-Shipley KMJ, Ford KL, Alberti F, Banks AM, Bailey AM, Foster GD. The good, the bad and the tasty: The many roles of mushrooms. Stud Mycol. 2016;85: 125–157. 10.1016/j.simyco.2016.11.002 28082758 PMC5220184

[2] Ford KL, Baumgartner K, Henricot B, Bailey AM, Foster GD. A native promoter and inclusion of an intron is necessary for efficient expression of GFP or mRFP in Armillaria mellea. Sci Rep. 2016;6: 29226 10.1038/srep29226 27384974 PMC4935854

[3] Bailey AM, Alberti F, Kilaru S, Collins CM, de Mattos-Shipley K, Hartley AJ, et al Identification and manipulation of the pleuromutilin gene cluster from Clitopilus passeckerianus for increased rapid antibiotic production. Sci Rep. 2016;6: 25202 10.1038/srep25202 27143514 PMC4855138

[4] Badalyan SM, Szafranski K, Hoegger PJ, Navarro-González M, Majcherczyk A, Kües U. New Armenian wood-associated coprinoid mushrooms: Coprinopsis strossmayeri and Coprinellus aff. radians. Diversity. 2011;3: 136–154. 10.3390/d3010136

[5] Banks AM, Barker GLA, Bailey AM, Foster GD. Draft genome sequence of the coprinoid mushroom Coprinopsis strossmayeri. Genome Announc. 2017;5: e00044–17. 10.1128/genomeA.00044-17 28385829 PMC5383877

[6] Gill-Carey D. Surface and submerged growth of Coprinus. Br J Exp Pathol. 1949;31: 30–35. PMC2073338 15420341

[7] Reina M, Orihuela JC, González-Coloma A, de Inés C, de la Cruz M, González del Val A, et al Four illudane sesquiterpenes from Coprinopsis episcopalis. Phytochemistry. 2004;65: 381–5. 10.1016/j.phytochem.2003.10.023 14759528

[8] Gonzalez Del Val A, Platas G, Arenal F, Orihuela JC, Garcia M, Hernandez P, et al Novel illudins from Coprinopsis episcopalis (syn. Coprinus episcopalis), and the distribution of illudin-like compounds among filamentous fungi. Mycol Res. 2003;107: 1201–1209. 10.1017/s0953756203008487 14635768

[9] Kettering M, Valdivia C, Sterner O, Anke H, Thines E. Heptemerones A-G, seven novel diterpenoids from Coprinus heptemerus: producing organism, fermentation, isolation and biological activities. J Antibiot (Tokyo). 2005;58: 390–6. 10.1038/ja.2005.49 16156515

[10] Johansson M, Sterner O, Labischinski H, Anke T. Coprinol, a new antibiotic cuparane from a Coprinus species. Zeitschrift fur Naturforsch—Sect C J Biosci. 2001;56: 31–34. 10.1515/znc-2001-1-205 11302209

[11] Abraham W-R. Bioactive sesquiterpenes produced by fungi are they useful for humans as well. Curr Med Chem. 2001;8: 583–606. 10.2174/0929867013373147 11281843

[12] Chadwick M, Trewin H, Gawthrop F, Wagstaff C. Sesquiterpenoids lactones: benefits to plants and people. Int J Mol Sci. 2013;14: 12780–805. 10.3390/ijms140612780 23783276 PMC3709812

[13] Surup F, Hennicke F, Sella N, Stroot M, Bernecker S, Pfütze S, et al New terpenoids from the fermentation broth of the edible mushroom Cyclocybe aegerita. Beilstein J Org Chem. 2019;15: 1000–1007. 10.3762/bjoc.15.98 31164938 PMC6541320

[14] Rasser F, Anke T, Sterner O. Terpenoids from Bovista sp. 96042. Tetrahedron. 2002;58: 7785–7789. 10.1016/S0040-4020(02)00943-2

[15] Wawrzyn GT, Quin MB, Choudhary S, López-Gallego F, Schmidt-Dannert C. Draft genome of Omphalotus olearius provides a predictive framework for sesquiterpenoid natural product biosynthesis in Basidiomycota. Chem Biol. 2012;19: 772–783. 10.1016/j.chembiol.2012.05.012 22726691 PMC3383649

[16] Quin MB, Flynn CM, Wawrzyn GT, Choudhary S, Schmidt-Dannert C. Mushroom hunting by using bioinformatics: Application of a predictive framework facilitates the selective identification of sesquiterpene synthases in Basidiomycota. ChemBioChem. 2013;14: 2480–2491. 10.1002/cbic.201300349 24166732 PMC3866635

[17] Medema MH, Blin K, Cimermancic P, de Jager V, Zakrzewski P, Fischbach MA, et al antiSMASH: rapid identification, annotation and analysis of secondary metabolite biosynthesis gene clusters in bacterial and fungal genome sequences. Nucleic Acids Res. 2011;39: W339–46. 10.1093/nar/gkr466 21672958 PMC3125804

[18] Weber T, Blin K, Duddela S, Krug D, Kim HU, Bruccoleri R, et al antiSMASH 3.0—a comprehensive resource for the genome mining of biosynthetic gene clusters. Nucleic Acids Res. 2015;43: W237—243. 10.1093/nar/gkv437 25948579 PMC4489286

[19] Blin K, Medema MH, Kazempour D, Fischbach MA, Breitling R, Takano E, et al antiSMASH 2.0—a versatile platform for genome mining of secondary metabolite producers. Nucleic Acids Res. 2013;41: W204–12. 10.1093/nar/gkt449 23737449 PMC3692088

[20] Saitou N, Nei M. The neighbor-joining method: a new method for reconstructing phylogenetic trees. Mol Biol Evol. 1987;4: 406–425. 10.1093/oxfordjournals.molbev.a040454 3447015

[21] Tamura K, Peterson D, Peterson N, Stecher G, Nei M, Kumar S. MEGA5: molecular evolutionary genetics analysis using maximum likelihood, evolutionary distance, and maximum parsimony methods. Mol Biol Evol. 2011;28: 2731–9. 10.1093/molbev/msr121 21546353 PMC3203626

[22] Afgan E, Baker D, van den Beek M, Blankenberg D, Bouvier D, Čech M, et al The Galaxy platform for accessible, reproducible and collaborative biomedical analyses: 2016 update. Nucleic Acids Res. 2016;44: W3–W10. 10.1093/nar/gkw343 27137889 PMC4987906

[23] Rutherford K, Parkhill J, Crook J, Horsnell T, Rice P, Rajandream MA, et al Artemis: sequence visualization and annotation. Bioinformatics. 2000;16: 944–5. 10.1093/bioinformatics/16.10.944 11120685

[24] Carver T, Harris SR, Berriman M, Parkhill J, McQuillan JA. Artemis: an integrated platform for visualization and analysis of high-throughput sequence-based experimental data. Bioinformatics. 2011;28: 464–469. 10.1093/bioinformatics/btr703 22199388 PMC3278759

[25] Townsend CA. A “Diels-Alderase” at last. ChemBioChem. 2011;12: 2267–2269. 10.1002/cbic.201100431 21796752

[26] Byrne MJ, Lees NR, Han L-C, van der Kamp MW, Mulholland AJ, Stach JEM, et al The catalytic mechanism of a natural Diels–Alderase revealed in molecular detail. J Am Chem Soc. 2016;138: 6095–6098. 10.1021/jacs.6b00232 27140661

[27] Kasahara K, Miyamoto T, Fujimoto T, Oguri H, Tokiwano T, Oikawa H, et al Solanapyrone synthase, a possible Diels-Alderase and iterative type I polyketide synthase encoded in a biosynthetic gene cluster from Alternaria solani. Chembiochem. 2010;11: 1245–1252. 10.1002/cbic.201000173 20486243

[28] Zhu Y-C, Wang G, Yang X-L, Luo D-Q, Zhu Q-C, Peng T, et al Agrocybone, a novel bis-sesquiterpene with a spirodienone structure from basidiomycete Agrocybe salicacola. Tetrahedron Lett. 2010;51: 3443–3445. 10.1016/j.tetlet.2010.04.128

[29] Zhang Z, Jamieson CS, Zhao Y-L, Li D, Ohashi M, Houk † K N, et al Enzyme-catalyzed inverse-electron demand Diels−Alder reaction in the biosynthesis of antifungal ilicicolin H. 2019 10.1021/jacs.9b02204 30905148 PMC6585442

[30] Ohashi M, Liu F, Hai Y, Chen M, Tang MC, Yang Z, et al SAM-dependent enzyme-catalysed pericyclic reactions in natural product biosynthesis. Nature. 2017;549: 502–506. 10.1038/nature23882 28902839 PMC5679075

[31] Chen Q, Gao J, Jamieson C, Liu J, Ohashi M, Bai J, et al Enzymatic intermolecular hetero-Diels−Alder reaction in the biosynthesis of tropolonic sesquiterpenes. 2019 10.1021/jacs.9b06592 31461283 PMC6944466

