# Supplementary material for: Bovistol B, bovistol D and strossmayerin: Sesquiterpene metabolites from the culture filtrate of the basidiomycete Coprinopsis strossmayeri
Source: PLoS One. 2020 Apr 6;15(4):e0229925. doi: 10.1371/journal.pone.0229925 (PMC7135263; doi:10.1371/journal.pone.0229925)
Supplement: S1 File — (PDF) [file pone.0229925.s001.pdf]

Supporting Information

Bovistol B, bovistol D and strossmayerin: Sesquiterpene metabolites from the culture filtrate of the basidiomycete *Coprinopsis strossmayeri*

Short title - Sesquiterpene metabolites from *Coprinopsis strossmayeri*

Alice M. Banks[1,#a], Lijiang Song[2*], Gregory L. Challis[2], Andy M. Bailey[1*] and Gary D. Foster[1*]

[1]School of Biological Sciences, University of Bristol, Bristol, UK

[2]Department of Chemistry, University of Warwick, Coventry, UK

[#a]Current address: School of Natural and Environmental Sciences, Newcastle University, Newcastle upon Tyne, UK

[*]Corresponding authors

Email: Gary.Foster@bristol.ac.uk (GDF); Andy.Bailey@bristol.ac.uk (AMB); Lijiang.Song@warwick.ac.uk (LS)

S1 Text. Internal transcribed spacer region of *Coprinopsis strossmayeri*.
>CBS177.39_ITS
GTTGTAGCTGGCTCTACCCGAGCATGTGCACGCCCCGTCACCTTTATCTTTCCACCTGTGCACA
CACTGTAGATCTGGATACCTCTCGTCGAAAGGCGGATGCGAGGCTTGCTGTGCCCTTCCCAAAG
GGTCGGCTTGCTTCGAATTTCCAGGTCTATGAACCCCTTTACGTACCCCAAACGAATGTTAAGGA
ATGTAATCATAAGGCCCTAGTGCCTATAAACCTATACAACTTTCAGCAACGGATCTCTTGGCTCTC
GCATCGATGAAGAACGCAGCGAAATGCGATAAGTAATGTGAATTGCAGAATTCAGTGAATCATCG
AATCTTTGAACGCACCTTGCGCTCCTTGGTATTCCGAGGAGCATGCCTGTTTGAGTGTCATTAAA
TTCTCAACCTCACCAACTTTTGTTGTGTGAAGGCTTGGATTTGGAGGTGTGCAGGTCCACATTTT
TTTAGTGGTCTGCTCCTCTGAAATGTATTAGTGGGTTAGGCCCCCTAATCTATTGGTGTGATAATT
ATCTACACCGTGGATTTGGGAAAGCTACATTTAGACCTGCTTCTAACTGTCCTCACAGGGGACAA
CATTTGACAATTTTGACCTCAAATCAGGTAAGACTACCCGCTGAACTTAAGCATA

S2 Text. Terpene cyclase protein sequences used in phylogenetic tree construction.
>Omp1
MKYTSFALPDLASSCDYNLRFNKYHRSVSPETKKWFFRLSPASQADLTTYDAQRFTLLAAVCYPDAG
YPQLRVCSDFLAYLFYLDNLTDDMDDKSTRSVADLVLNSLNEPETFQTQYRIGKMTSDYFKRIIQTSN
DGTKKRFIDTMDSFFKSVDDQARDRLAGHIPDLESYIALRRETSGCKTCFSLIEYANNLHIPDEVISHP
HIEQMETAANDVVSFANDIYSFNIEQSKGDTHNMIPVLMHANPDMDFLEAVSFVRDLTIKAMDRFNEL
RATLPSWGLDIDKDMKVYVNGLENWMVGILFWSFETERYFGKSVRSVKATKTVNLLPSRA-

>Omp2
MASTAPSKFILPDLVSHCSFDLHHNRHRKQITTETKKWLFKGDNLTGRKRDQYHGLKCGLLSAMCYP
NAAYPQLRVCNDFLTYLFHLDNLSDDMDNRGTTTTADVVLNSLYHPGYFQSARVGKMTRDYWKRLI
STASPGTQQRFIETFDFFFQSVTEQAHDRQAGVIPDLESYIALRRDTSGCKTSFVLIEYANNLDIPDGV
MDHPLIRSLGEAANDLVTWSNDIFSYNVEQAKGDTHNMIPVIMNEHGLDLQSAVDYVGRLCQQSIDR
FISDRAQLPSWGPEIDRQVAIYVDGLTDWIVGSLHWSFESERYFGKSGRQIKKSRVINLLPRRA-

>Omp3
MAIENTIASAPASTPAKQLDTPDHFILPDLVSHCTFPLVYHSNGDAVAAQSVKWLDTNCPDLNDKRRK
ALYGLQAGELTAYCYNTAPDQRLRVVSDFMNYLFHLDNISDGMMTKDTDALSDAVMNALWFTEWYR
PTKKSDYVQPDEELNAGKLARDFWHRCIQDAGPGCQARFKETLELFFEAVNIQAKARDAGVIPDLES
YIDVRRDTSGCKPCWALIEYGLGIDLPDYVAEDPIIKSLNQSTNDLVTWSNDIFSYNVEQSRGDTHNMI
VILMLYHGHNLQSAIDYVGDLCRQTIDDFKENRKKIPSWGPEVDDIVKQYVQGLQDWIVGSLHWSFM
TTRYFGKQGQEVKKNRYVKLLPVGEEANKW-

>Omp4
MSSAPTRFLLPDLLSACPLKGSVNPYYKEAGAESSAWINSYDIFTDRKRAFFVQGNELLVAHTYPYAG
YEEFRTCCDFINVLFVLDEVSDEQSGSDARFTGEVFLNALRNPENDDTSKLSKISKEFRARYFKRAGP
RTAERFLQHCQDYIDCVTREAELRERGEVLDLPSFTALRRENSAIRVCFCLFEYALGFDLPQEVFDDP
TFMEMYWAAADLVCWANDVYSYNKEQAQGHGGNNIVTVLMKAKDLDLQAACDYIGVYCEELMGRY
LSAKARLPSWGPEVDAAVAQYVEASGHWVRGNLDWSFETQRYFGAQHAEIKETRLVTLTPAIPEDF
SDTGSESE-

>Omp5a
MSPDPTRIVLPDFLAACPFESSTKNPHLKAAGAESSAWVNSHVVFNDRKRAAFMQDIYELLVAYAFP
WADYEDFRTMCDFINLLFVLDELSDDQNGKDAGYTGKLFMDAMRNIDNGDTSELTELCREFKARYSK
RVSPQVNERFLQHLQSYTDCVAQEADLRERGEILDLESYVALRRENSAIRPCFDLVEYIIDFDIPQEVID
HPVFSEMYWASVDLVCWSNDVYSYNVEQAKGHGGSNVVTVLMKEKNLDLQAACDYVGVYYEELM
DRYLSAKARLPSWGPEIDAAVGKYILAEAQFVRGNLDWSFDSPRYFGPQHDQVKKTGIVTLTPAPKK
FGSDSGSESE-

>Omp5b
MSPAPSRIVLPDFFASCPFESSTINPHFKAAGAESSAWVNSHVVFNDRKRAALMQNSYELLVAYAFP
WASYEDFRTLCDFINLLFVFDEVSDDQNGKDAGYTSKIFMDAMRNIDNGDHSELTELCKEFKARFSR
RLSPQVNERFLQHLQSYTDCVAQEADLRERGEILDLESYVILRRENSAVRPCFDLVEYIMDFDIPQEVL
DHPVFSEMYWASVDLVCWSNDVYSYNVEQAKGHRGSNVVTVLMNEKNLDLQAACDYVGVYYQEL
MDRYLSAKARLPSWGPEIDAAVGKYVLAEAQFVRGNLDWSFDTPRYFGPQRDQIKKSRIVTLTPAPK
KFGSDSGSESE-

>Omp6
MIAKNSEIDRFYIPDTLANWPWPRHLNPAYPEAKKASAAWLRSFNAFNERSQKAFDLCDFNLLASLAF
PLADLYCLRSGCDLMNCFFIFDEYSDVADPQTVRQQADIIMDAIRNPHVPRPRGEFIGGEAHRQFWE
RAMQGATPTAQRRFIDTYQQYTDAVVQQATDRADNHIRDVEGYFTVRRDTIGAKPSFTLLEFTMDIP
DEVMGHPVIKDLSLWCIDMLIIGNDLCSYNVEQAHGDDLHNLVTIVMNQYNLDLPGAMEWIGKFHDDI
ADKFLDTFAKLPSWGPEIDPQIRRYVDGLGNWVRGNDSWSFESWRYFRGKGPEIEKTRWVDLMPT
EEATITPKYESDSNAAQPAQST-

>Omp7
MPETFYLPDCLANWKWKRALNPNYPEVKAASSEWLRSFKAFPPKAQEAYDRCDFNLLASLAYPLAD
KDGLRTGCDLMNMFFVFDEYSDVAHESEVQVQADIIMDALRNPHKPRPVGEWVGGEVTRQFWELAI
KTASPQSQKRFIETFDTYTKSVVQQAADRTQHYVRTVDEYLEVRRDTIGAKPSFAILELTMDIPDEVIH
HPTIERLAILAIDMILLGNDTASYNYEQARGDDNHNMVTIVMHQYKTDIQGALSWIEKYHKELEEEFMQ
LYNSLPKWGGQIDVDIARYVDGLGNWVRASDQWGFESERYFGTKAPEIQKTRWVTLMPKKRAEGV
GPEIVDISEL-

>Omp8
MSQILHLLWSKFSTSLPSTVTIGSDPQTLQLVHSPAPNVNANALEIRRIVNNFLTRCSIRLEGTPLDVDF
YNECKKTLLSHYIGIHDSDKVSESWFRRYLSVGVIITTNAYGHIDDKPTRVYICLYTALLTSFDDVFEAN
VEQFGGFNERFMKGEPQEDLFLDALARILLDAPRYYGRLATNIIVTATLDFFTGLFLELQARDMTFNED
LHNFAVFCRNLTGIAHAYAVFMFPRDVPFTVYVRSLPELKTDINYVNDIMSFYKEDRAEETDNLASILR
QVHPSMTKHQVLQKIVDDAVEADVRARKILADYQPALDAYEHFRKGYAMFHVSSGRYRLDELFSYIR
FE-

>Omp9
MSQILHLLWSKFSTSLPSTVTIGSDPQTLQLVHSPAPNVNANALEIYKIVDNFLSRCGIRLESTPLDVEF
YNECKKTLLSHYIGIHDSDKVSESWFKRYLSVGVIITTNAYGHLDNKLTKIYIALYTALATCFDDVFEKN
VDHMSGFNERFMKALPQGDVFLDAFAKVLLDAPKYFGRLASNIIVTSTLDFITSMSVDVLTKGMKFNQ
NLHKFAMACRNMSGIAYTYAPFIFPKEVPFAIYAQCLPDMRIYINHVNDVLSFYKEDKAGETENLASIL
GQVHPSMTKYQIVQGLADDAAEADLRVRTVLSQYQPALDAYNCFRQGYVSFHASSGRYRLDELFSF
VEPEPIV-

>Omp10
MTLPTEQVELSVCPVESSTHTTRDIMRNFLSQCQIPLQRGVPLDPTFHQECANVLIEDYLKPSAAVTL
ENLPSLMSSFNPFLTLGVRMASTGYAHLTHTPTRVYVALFTALLVCLDDIFPENVELMCGFNERFIKNE
TQGEPILDAVAGLLRSTSKYFSMLSSNLIVTSALNYVTSLSLDQGLHSIKLAEHSRNFARLCRNMSGIP
EAFAAFVFPPEVPFTAYIQCFPDLYTYANYVNDVLSFYKEDIAGETENLVSILAQTQPNSSRYQVLQQL
ADEAAAANANIRDILSDQKSILDAYDAFRVGFVQFHIDSPRYRLAELFPCIDG-

>Cop1
MSSLDATIHPVLNFEDKKIVLPDLVSHCNFKLRVSRHRKRITGETKRWLFKGDNLVGPARNKYHGLKA
VGRMTRDYWRRMILTASPGSQQRFIETFDFFFQSVTQQAIDRLTGEIPDLESYIALRRDTSGCKPCWA
LIEYANNLDLPDEVMDHPVVRSLGEAANDLVTWSNDIFSFNVEQSKGDTHNMIPVVMHQEGLDLQSA
VDFVGEMCKSAIDRFIEDQNYLPSWGPKIDRDLAVYINGLADWIVGSLHWSFETERYFGKNGRQVKS
SRVIDLLPRRSQ-

>Cop2
MPSPAGALPKSFILPDLVNDCPFPLRVNPLCDEVGRLSEQWFLRHANYSPPRAVAFMALKAGELTAA
CYPDADAFHLRVSDDFMNFLFNADDWLDDFDIEDTYGLANCTVRALRDPVNFITDKRAGLMTKSYFS
RFLKTAGPRCTERFIQTLALYFESVVTQKQARNNGTLPDLESYITIRRNNSGCKPCYALIEFCAGIDLP
DEVINHPIIQSLEDASNDLIAWSNDIFSFNREQSRHDSFNMVSIVMHQKGFALQEAVNFVGELCKKAM
ERFQADKRNLPSWGPEIDGEVAMYVDGLQNWIVGSLNWSIDGTERYFGKDGPGIKKHRKVKLFPKR
PLKTPAVRVLA-

>Cop3
MSTPSSSLTTDESPASFILPDLVSHCPFPLRYHPKGDEVAKQTVHWLDSNCPDLTAKERKAMYGLQA
GELTGYCYPYTTPERLRVVADFLNYLFHLDNISDGMMTRETAVLADVVMNALWFPEDYRPTKGQAA
EELNPGKLARDFWSRCIPDCGPGTQARFKETFGSFFEAVNIQARARDEGVIPDLESYIDVRRDTSGC
KPCWVLIEYALGIDLPDFVVEHPVIAALNQGTNDLVTWSNDIFSYNVEQSKGDTHNMIIILMEHHGHTL
QSAVDYVGSLCQQTINTFCENKQQLPSWGPEIDDMVAKYVQGLEDWIVGSLHWSFQTRRYFGDEG
QEIKQHRLVKLLTVAPPPPPPPPPTPPPQSSDADTKKQKVKAQDGKGPVSDEEVWALVRAEQSKGSIL
ESLFGFLTTSLSRIFFGYFFAYSH-

>Cop4
MRPTARQFTLPDLFSICPLQDATNPWYKQAAAESRAWINSYNIFTDRKRAFFIQGSNELLCSHVYAYA
GYEQFRTCCDFVNLLFVVDEISDDQNGQDARATGRIFVNAMRDAHWDDGSILAKITHEFRERFVRLA
GPKTVRRFADLCESYTDCVAREAELRERNQVLGLNDFIALRRQNSAVLLCYSLVEYILGIDLDDEVYE
DPTFAKAYWAACDFVCWANDVYSYDMEQAKGHTGNNVVTVLMKEKDLSLQEASDYIGRECEKQMR
DYLEAKSQLLQSTDLPQEAVRYIEALGYWMVGNLVWSFESQRYFGAQHERVKATHVVHLRPSSVLE
ASCDSDSDSDC-

>Cop5
MVGSYTGKVIHVPALLESWPWPAAINPLYEQVQEESTSWFRKFDLYRDRKKQAIHDHLDTAKFGASV
CPKADYALLRLATDYLHLGFWIDYFFDTSPSDVIRQLTESIAHLLESGDPRLDSSSPQSHIACMEILRDF
RKRIETFNPSQEDLRRFVKEYRGFLEAELTQAIDHENKVIRDIESYLSIRRSTIAIRPGIALLGLALGIPQE
ILDDPYTDTLTNACLDMVIIQNDAYSWNVEQVRKADGHNIITVLMKQRDIDVQEAYEHAAQLHRETQE
HFLELHAKRPDWGNEGSIQAFFDGLGEFVRGVDEWSSMCLGEHALSVGAGFLK-

>Cop6
MPAALPYNVSRDNKWDIKKIIQDFFKRCDVPYQVIPYDTELWNACLKRAKEKGYPVEPDSPMSLYRS
FKVGVVITRTSYGHIQDYEILIWVATFTAFVTYADDAFQEDIQHLHSFARTFLQNEKHEHPVLEAFAQF
LRESSIRFSHFVANTVVSSALRFMMSIALEFEGQNVSVSTEAREYPGYIRILSGLSDIYALFAFPMDLPR
STYIQAFPEQIDYINGTNDLLSFYKEELDCETVNFISAAATSQQVSKLEVLRNAAEKAAYSYDVVVNVL
KPYPEALAAWKSFARGFCYFHTSSPRYRLGEMFHDFEHDLVCKCASCTEI-

>Fompi1|63546
MTSTFYIPKTMANWPWQRAINPHYKEINAKSNAWLKTFKPFNEKSQIVFDKCAVEHLRTGCDLMNLV
FILDEYTDVEDANVVREMVDIVIDAIHNPEKPRPEGEVLLGEITRQFWALGIQTCTVTARKHFEEAFTD
YVISVYDQALNRATKSIHTVETYFKARRENIGIRPSYIPAVLGIDIADEAFYHPMVVELAYLIAVLVVLDN
DIYSYNKEQATGDDQYNIITIVMNQYGYTLDEAMKWTANCHEEVEARFMKGMKELPYFGPEVDPQLQ
QFIQALALWPRANDCWSFESGRYFGSRGLEIQKTRTVPTMPKVVDNRQSLRRENVVIPLID-

>Fompi1|84975
MSTWPWPRRIHPCAEEAVAASRDWLRTLHAFNEESQRAFDKYNVGLASALTYPFSSQELLRIGCDLY
NLFFVFGHYTDVEDAQACRTMADITMDALYCPYTPRPQDEVALGEITRQFWLRVIESGIVGPVAQRR
FIDYFTAYTDSVVEEASDRDAGVYRQVEDYFAIQRDTLGFRPLYALMVLELPDEVVYHPAIVEMVICVI
DMLIIDNDLISYNMEQRSGDARHNLVTLTMLEQTLSIHDAITLLFKRHISFQDRFLKAYRSFEPQWDDAI
NAQLRDALRDLAHFPRGIYCWHFGCGRYFGHKGAEVSVSRDVELLPQVLGARDLKRENVKLLVMDE
F-

>Fompi1|112148
MTIPEVSRSPPDHFVLPDLVSHCKFPLSVHPNALAVAANSDRWIDKGCPELSPEKRHAIYTLKAGILTA
QCYPHCDDEHMRVVADFLVFLFHLDNMTDVMLAAGTEQLADLVMNAHWFPEKYTPTHATGKEQPD
EEPSAGKLARDYWSRCTKDAKPGPQARFKQNLDLFFSAVQIQAGDRSAGIIPDIESYIIDRRDTSGCR
PAFDLIEYSMGIDLPEYVLEDPSIVALSNASNDLVAWSNDLFSYNVEQARGDEGHNMITIVMHHQGRG
LQEAVDYVGELCRQTINTFADNLECIPSWGPEIDRDVKIYVKGLQDWIVGSLHWSFITHRYFGSLGPEI
KKTRFVKLHPKKKPEPPL-

>Fompi1|85203
MSVPSPPESFVLPDLVAHCPFPLARQPNAKAIAAASDKWLDDGCPELSQRKRAALYGLQSGILTAHC
YPDADDEHLRVVADFLVYLFHLDNISDTMAVTGTEQLADVVMNAHWLPERYAPTTSPGKEQPTEEFS
AGKLARSYWSRCIEDAKPGPQARFKENLELFFEAVQLQTQDRDSGAIPDLESYINIRRDTSGCKPSFD
LIEYAMRLDLPDFVVEHPVIKALNQAANDLVTWSNDIFSYNVEQARGDTHNMIPILMIHHKLSLQEAVD

YVGNLCKQTIDGFIENEKRIPSWGKEVDKEVSVYVKGLRDWIVGSLHWSYMTERYFGATGSHVKESR
VMKLLPKRQRTA-

>Fompi1|53631
MSLSPKSFVLPDLIPLLPFKGSFNPHYERASKSSSAWVNGYKIVPDRKRAFFQQGGSELLCAYAYSY
AGYEELRTTMDLVNILFTVDEVSDEQNGKDAYKTGRVFLNSLRDPDWHDGSALAQMTKDFRKRLLQ
FNVPACYRRLLKHCEDYVNAFAVEAELRERDEVLDPESYIVLRRENSAVRFCFGMFGFTLGLDLPDEI
FEHPVMMRLHLAAVDMVCWSNDLYSYNMEQAMGHTGNNVMTVLMKSQQCDLQAAADHVGAHFK
QLMESFEADKARLPSWGPKLDAVVAKYVMAMETWVVGNCEWSFETQRYFGVQREEVKRTLVVKLY
PKHEENDD-

>Fompi1|88978
MSSPSKSFVLPDLISLLPYKGSFNPHYERASTSSSAWVNGYKIVPDRKRAFFQQGGSELLCAYAYSY
AGYEELRTTMDLVNILFTVDEVSDEQNGKDAYKTGRVFLNSLRDPDWHDGSALAQMTKDFRKRLLQ
FNVPACYRRLLKHCEDYVNAFAVEAELRQRDEVLDPESYIVLRRENSAVRFCFGMFGFTLGLDLPDEI
FEHPVMMRLHLAAVDMVCWSNDLYSYNMEQAMGHTGNNVMTVLMKSQQCDLQAAADHVGAHFK
QLMESFQVDKARLPSWGPELDAVVAKYVMAMETWVVGNCEWSFETQRYFGVQREEVRRTLVVKLY
PEDKDNDE-

>Fompi1|40901
MSKPASPRRFVLPDLPSLCPFQPSTNPHYTRGAAESRAWINAYDVFTDRKRAFFVQGCNELLVSYTY
PYACYEQFRTCCDFVNLLFVVDEVSDEQNGADARRTGEISLNVMRDPSWDDGSKLAKMTREFRARL
TAHAGPGCMRRFLKHFEDYIDAVAREAEYRERGEVLDMVSFEALRRENSAIRLCFGLFEFCFGVDLP
DGVFNDPTFMTLYWAAADMVCWSNDVYSYDMEQARGIAGNNIVTVLMHDKGVDLQTAANLVGEHF
KTLMACFVEAKTRLPGWGPTADEAVRKYVKAMEHWVTGNLEWSFETQRYFGPLHSEIKRTRIISLRP
REEPEYE-

>Fompi1|162166
MSSSLSASSPAPSKIIIPDLVSHCTIPVRCNRNWRLASVESKQWLFRGGDLTQKKRDAFHGLKAGYLT
SMCYPLAGYPQLRVCCDFMNYLFHLDNLSDEMDDRGTLGTAIKVMDPLYHPEASRPTTRVGKLTKD
YWRRLIQTGSPGAQQRFIETFDMFFQAITEQARDRANGLIPDLESYIAIRRDTSGCKPCWALIEYANNL
DLPWEVMDHPIVRGLGEAANDLVTWSNDIFSYNVEQSKGDTHNMIIVVQKQQGLDLQSAVDFVGDL
CKHSIDRFHFLRENLPSWGPEIDRDLAIYVDGLADWITGSLKWSFESERYFGKQGLEVKRTRVVTLLP
RRA-

>Fompi1|132954
MGSSQSVPSPPRHSRRISLPPPPKPSSSPAPELKGGEIIQFPDLVKSIPFPIRLNPYTRFASAESDAFII
EYGKFSEKQLNKFVGLNAGLLCGMCYAECEPEQLRVCTDFMSFLFNLDDWSDEFDTAGTKGLEEAV
MNTLWHPDTYHSDSVAARTAKSWWLRMLKTVGPNCRRRFVDTLELYFKAVMQQAADRTAKRVPEL
EAYVSLRRDTSGCKTGFALIEYAAGIDLPDEVVEHPVIQGLLDATNDSVSWANDILSYNREQSRGDSH
NIVCAVMATLGLERQAAIDYAGKLCTKTVERFLEGKAALPSWGPEIDAQVRTYIQGLEDWIIANAEWS
FMTERYFGKDGPRIRKTLQVALLPLKGFD-

>Fompi1|88169
MTTTPESYILPDLISLLPYQTPISPHYEQAAAESSAWLSSFSGVIPAHKRRFFEQHGSELLCGYAYSYA
DHEELRTAMDFVNLLYVYDDIGDDQNGQEASETGSALLNALRDPNWNDGSQLAQMAHEVQARLRQ
VDAPACYARFLKHCEDYISANVRQTELRERGEILELDEFIPLRRENSGVPLCIGVIGFVIGRDIPDEVFE
HPVMTRLYLAAVDMVWLANDLYSYSREQAISLSGENIMTILMTQESCDLQGAADRVGRRFADLIAGF
EADKARLPSWGVDFAAFVKGLETWVVGNCQWSLETQRYFGVHVEEVKRTRVVKLDRKRDVGMED
GSNDG-

>Fompi1|90088
MTAPPSTLRIAQEAVRSFLDNVDIHLPKYARDRELERRVNEVVSQWGISYDEMRAYVVPATVLTITAY
SHITDMDTKVLITIFTTFIAIMDDPVFFEGLTPADFHRNMCTGAVQGDSGLLGRFSRVLGSIWDHYPGF
TANTIYASALRYINASVMENEWHGEIFSREASLFVEYKRTMTATTEAYACFIWPASQFPDYKTYVQAIP
DTMTYVSRVNDILSFYKEKLAGEKDNYVHERALATGRPTSTVLQELITETNSAVRRVRYLLRDVDALA
AWENFAAGYIRVHTDTPRYRLKDILGGEYFIDVSGM-

>Fompi1|90084
MSLGSHLSAVKVSQDAIRRFLSKVNVEFPQYTRDAELEQRVKSIVCDWGDEQALRPYVITALILTITAY
AHVSDLETRVLITLFTTLIIAMDDPTVFNGLLPVEYHRKMCTGAVQQERGMLGEFTKVLQGMWDHYP
SFTANTIYASALRYVNASVMENEWRGEAYTQEARPFVEYKRSMTATTEAYACFIWPQAQFPDYRVY
VRAIPDTMFYVSYVNDILSFYKEELAGEEDNYIHERALATGSSASAALQEVIDDTIAAVKRVRSVLGNG
PARAAWENFAAGYIRVHTDNPRYRLKDILGGEYIIDMASY-

>Fompi1|90124
MPAQVIADPTPQKDTIAEVAKVAIWEFLQKMEVSVTPPRYERDVELEARVKATTHTWPLEHRLQRHIV
TALVITETSYTHVSDVEARVAIVLYTAILTAIDDPDLFDSVGAEDFWRRVCDGSLLQDRGIMGEFARVIL
SMGRFYSNYCSATILAASLRFLNGEMIGNPENASFVEPNSKEFVDFSRDLSGDAEAYAAFIWSKTEFP
NDTSYIQIFPDACIYINHANDILSFYKEEQDGEVASYIHARARVTGKPPIATLHEVIDEVVAAAERIRCTL
GNGKARKAWDSFERGYIGFHIGDPRYRLRDIFGHWEYMRDAGAV-

>Fompi1|114001
MVSSPSTLSPPATKEQISAVSREAIHNLIDGLHFTAFPASRDPELESRVVERIRSWGSDMLDLLRPYIP
AATILTITCYAHIDDMDIKVQIVLFSALVIAMDDPSVLSSPSVRDFHRRLCMGAAQDDAGVVGRLLQVL
LGEWDFYADFSATSIFTSVSQFVNGCLLEQISGEAQLACSSDALPFIAYRRNMSGLAEAFAYFIWDKK
RFPHVEAYMQAIPDICWYQNHANDVLSFYKEELAGETGNYMSDRARASGISIQDTLQDVVDETIVIVE
RIRSILGEGPVRDAWESFASGWIAYHTHSPRYRLKELIDCEYIIIDGISQHPPRPRDQKAVGVDGEALE
TAATKLHIAEEFYQ-

>Fompi1|55998
IREFVQSLQIPQPTYAYDSALRARIKEIVSAWDFIGNMRPQLLTGVTLAETSYNHITNVDTKAAIALYCA
LFAYIDDKKVFDAMVAQRFPRQLCCPSPDDSSFLVALRSVLLSMWDWYSDFGANTILYGGLEFINGS
MLESAPGDSQIIQARTLPLVELRRNMAGIPDPFLCFIWEKSVFPDETLYAEAIPDVRVYSDYINDILSFY
KEEMKGEVDNYVHARARITEKAVVETLHDVVRETVAAAERVRRTLGEGRARDAWDRFEQGFIRFHL
RDPRYRLQDVLGTHWDYLVPLL-

>Fompi1|84944
MSRRYQTSRFSPSAKGKKTSLYTSPGDLRALFHRFSLYAGVQLESFTSPKMMQIEVPVMLNLKESSM
PMNSTVLEDCTAAVAKDAIVQFLRRLGAVLRPSFGNNRDLEERVKEITKTWPFEHRIHPHITTGVVMA
NTTIAYLSDLDARAAVAAYTALITALDDPDIFHASGAQNFAQMLCDGSALRDDGVLGQMARVLADMG
NHFPPFGTSAIIAATLRWCNGELISNPANPFCLRPLSKAFADYQRGLTGVPEAYAAFGWCKADFPVET
DYIHVFPDICFFLNHTNDILSFYKETLDGESDSYIHARARLTGKSVTDTLYEVMDEVITTTERIRKHFGE
GRMRNAWDRYEAGYVWFHTGNPRYRLHELVDTEYMPMY-

>Fompi1|56121
MIMNFLRRMDFTLPEHFCRDTELAERVKAVTMTWPFEDRVRKHIVTGVACAEACYPHLDIDARTAIAI
YTSLTVIIDDKESFESLAGAEAFSQMLCDGTIHRDEGPLGQLVKVFTDFHSLFPPFHSSIIVASTLRFIG
GEMITNPCHPSFQEAHSKAVVDYQRWMTGVAEPYVLFNWPKAARTALFNGLPDCIIYINHGNDILSFY
KEELEGDTNNYIHARARVTNQSPYKILQDLIDEVVAAAQRIHSSLGDGPVRDAWDSFEAGFIGFHIGD
PRYRLREIIGSQYIMDHTTVYL-

>POSAmga1
MSQRIFLPDTLANWQWPRHLNPHYAEVKKASAAWAKSFRAFQTKAQEAFDRCDFNLLASFAYPLAD
EARLRSGCDLMNLFFVIDEYSDVSTEEEVRAQKDIVMDAIRNTEKPRPAGEWIGGEVSRQFWDLAKK
TASTQAQKRFIDTFDEYLESVVQQADRNNSHVRGIESYLEVRRNTIGAKPSFALLEFDMQLPDESHQS
SGYQRNLRKSCIDMLCLGNDVVSYNLEQARDDDGHNIVTIAMNELRTDVAGAMIWVDEYHKQLESRF
MENFKKVPRWGGPIDLQVARYCDGLGNWVRANDQWSFESERYFGKKGPEIIQRR-

>C.s_contig36
MADYDNILTRLSQKPQWSKESEAVTILEPFTFTTSSPGKEIRGKLIDAFNLWLNVPGDKLQVITKIVNML
HAASLMYEDYIEDDSQLRRGTPVAHKIYGIPQTINSANYVYFLAFQELFALRNAPTPPRQDIDQLVTHE
LLSLHRGQGLEILWRDSLQCPTEEEYIDMVKTGGLLRIGIKLMMACSTTNSDIDYTPLISLFGVYFQIRD
DLMNLQSPEYTSNKGFAEDLTEGKFSFPVVHGIHADRSNRQVLNVLQKRPATPTLKIHTINYLQNHTK
SFEYTLTVLKNLEGQLRREIAKLGGNEKFERIVDLLHVDEEAFSSKQHR-

>C.s_contig56
MRPSANQFTLPDLFSICPLQDATNPWYKQAAVESRAWINSYNIFTDRKRAFFIQGSNELLCSHVYAYA
GYEQFRTCCDFVNLLFVVDEISDDQNGRDARATGQIFVQAMRDSQWNDGSILAKITHEFRNRFVRIA
GPNNIRRFAELCESYTDCVAREAELRERGEVLGLNEFIALRRQNSAVLLCYSLVEYILGIDLPDEVFDD
PGFSKAYWAASDFVCWANDIYSYDMEQSKGHTGNNVVTVLMKEKGLSLQEASDYIGQQCKIQMEEY
LLGKVQLSPDLPPEALRYIEALGHWMIGNIWSFETQRYFGANHQRVKATRVVYLRPYKVLEASDSDS
DSD-

>C.s_contig69
MGAINWLVLLLTHPFEFRTLVQYWLYHEQKRDIKALQEHPTSGWDRESMQRCWQFLDDTSRSFAAV
IKELDGDLARTICMFYLVLRGLDTIEDDMTIADHVKQPLLRAFYEHTVTPGWKFDGCGPNEKDRQLLV
EYDTVVEEVNLLAPHYKSVIIDICHKMATGMADYAHKAATTGSIYLDSVAEYDLYCHYVAGLVGEGLS
RIFSASGKEQEWLGEQLELSNSMGLLLQKTNIIRDYREDTDDQRHFWPKEIWGQFGFNDVKELYQPE
NAEGAQWAQSAMILDALRHAEDGLDYLRLLKNQTVFNFCAIPATMAIATLELCFMNPAMFQRNIKIRK
AEAASLIMRSTNPREVALIFRQYVRRIHAKAVPSDPSFLKISVACGKIEQWYEHNYPSFIKVVESPEGP
RPEIDAEDVRSRTFSAITARNKQLEKRQKQNGANGNAAAAAFPQVATQEATTGELMMYVAAAIGVVL
VLSGGIVYLVIQLSGESSPLRGTSSGTGFGEL-

>C.s_contig81
MSSSQSFNEKPTSFILPDLVSHCRFELSYHPSGDAVAAQSVKWLDDNCPDLDAKQRRALYGLQAGE
LTAFCYNNTTPERLRVVSDFMNYLFHLDNISDGMMTRETDVLSDVVMNALWFSENYRPTSSQPTEEL
NPGKLARDFWARCIPDCGPGAQARFKETLGLFFEAVSIQARSRDEGVIPDLESYIDVRRDTSGCKPC
WALIEYALDIDLPDFVVEHPVIEALNQGTNDLVTWSNSRGDTHNMIVILMEHHGHTLQSAIDYVGDLC
QQTIDTFVVYNKNNLPSWGPEIDDMVARYVKGLQDWIVGSLHWSFQTHRYFGKEGQAIKQHRLVKLLP
PVDSTPPPPPPPTTRAPTPTPAPKGRKSRENGLYRGRLADDEVWALVRAQPQEPRFISWVTRVLGH
LPRLFFGFLFPYSH-

>C.s_contig98
MPGSADWTPDRFYLPDTLANWPWPRAINPAYDECKAASAAWCAKYGAFSARAQKAFDLCDFNLLA
SLAYAHLPADVNRVGCDLMNLFFVVDEHTDAMDARSVQEWVDIVVDALHNPHKPRPAGEPIVGEVA
RTFWENGVKCLGPTSRSRFVETFTTYLQSVVTQAQDRDNHLFRDVNSYMDVRRDTIGAKPSFALLE
HDMEMPDEVFNHPLLENLREWAIDMLVIGNDLCSYNVEQSRNDDGHNIVRLAMLQENTNIHGALRFV
SNMHDDLVQKFLDNYKNMPSFGQLIDEWVSRYIEGLGNWVRANDTWSFESWRYFKGDGLRVQEER
WVDLLPPAPKDELTSSSTLFFTRPLYPGFLHCC-

>C.s_contig109
MYFVFPDLVSDCPFPLRINPHCSKAARASEKWLLKYANLPLARVVAFKGLKAGELTAACYPNASMFH
LRVCDDFMNYLFNLDDWLDDFDVEGTYGLAKCCLAAMRDPFRFVTDKRAGLMTKSFFQRFVKSAGR
RCMDRFIHNMDLFFQSVIIQSQNRAQGTIPDIETYITIRRDNSGCKPCFQLAEFAAGIDLPDEVIQHPVI
QSLEEASNDLVTWSNDLFSFNVEQSRNDTFNLVCVVMYEKDYSLQEAVNFVGELCRKTIARFNSEKE
NLPSWGPERDQQVAMYVDGLQNWIVGSLHWSFDSERYFGTQGHEIKKHRKVELLPKRPS-

>C.s_contig158_GGS
MYRCSATRNGLRNVFSRYRRRTYSTKGPDPFKLVEPQLNELRASLLSLLGSGHAAISEVTKYYFLHP
SKHVRPVIVLLLSQATNGLGSDWNKKLWEAQNTGGGGREEDLNVPLSRPDVLNDWNPAMPSGTSP
FHTTFPLQPLKPHRQPASKPLHLPSSPSNSPSLHSPPFVLPTQVRLAQIVEMLHTASLLHDDVIDESAL
RRGAPSAPSAFDNKQSVLGGNFVLGRASAALSRLGDTEVTQLIASVISNLVEGEILQLKEIKLDGSPES
VNQTRQDAWNIYLHKTYLKTASLMAKGARSAVVLGGCKEGEIWKEIAYAYGRNLGIAFQLVDDVLDY
ESASATLGKPGGADLELGLATGPALYAWEEFPEIGELIRRKFEQPGDVERARELVLRSSGVERTKALA
QAYAAKAREVLQEIPDSEAKAALEMGALPRPHERLMSDCNRQTHQQL-

>C.s_contig158_Cyc
MPYQSTYPSVESFASDETHIDNVLQSSYPVIMVEDEKKSDPHFISPLPALPYLKAGSFPESPFNNAPA
SPYLNRTSNFDLDSGWRRRQAIQRGEKRKVKLTQGHFIAEYRVPTAVHNAMEKRYTATKSTEFSHM
RYTAATCDPDDFTMENGWTLRPRIYHRQTELLIAVTSYNEDKALYARTLHGVMLNIRDICKTQKSKYW
RSCAEEGTPAWQKITVALVVDGLDNMDKSVLDILATVGVYQDGIMKKKVDGRDTVAHIFEYTTQLSVD
AKPQLVLPQENDDGSNLVPVQIILVVKAKNQKKINSHRWLFNAIGRQLNPEICVLVDAGTKPGYKSIYH
LWEAFYNNENLGGCCGEIYAMGGKRLLNPLVAAQNFEYKMSNILDKPFESSFGYVSVLPGAFSAYRY
RAIQGRPLDQYFHGDHSLATRLGDKGINGMSIFQKNMFLAEDRILCFELMAKRGEKWTLGYVKNSKA
ETDVPESAAELIGQRRRWLNGSFAASIYALVHFWRVYQSGHNFIRIFFFHIQALFNAFSLFFSWFALAN

LWLTFSIIIDLLPATIKGANLEVFHWVHSVLKYIYLGFLAMQFILALGNRPKSERVAYTTSFYVFSALSLY
LIFASLWLTASSFMNLESDIARHNYKSSLEAVKGMFETPIGPLTAAIVSTFGIYLIGSILYLDPWHMIHSF
LQYFILAPCFTNILNVYAFCNLHDVSWGTKGSDTVD-

>C.p_cyc1
MRIPNVFLSYLRQVAVDGTLSSCSGVKSRKPVIAYGFDDSQDSLVDENDEKILEPFGYYRHLLKGKSA
RTVLMHCFNAFLGLPEDWVIGVTKAIEDLHNASLLIDDIEDESALRRGSPAAHMKYGIALTMNAGNLVY
FTVLQDVYDLGMKTGGTQVANAMARIYTEEMIELHRGQGIEIWWRDQRSPPSVDQYIHMLEQKTGG
LLRLGVRLLQCHPGVNNRADLSDIALRIGVYYQLRDDYINLMSTSYHDERGFAEDITEGKYTFPMLHS
LKRSPDSGLREILDLKPADIALKKKAIAIMQDTGSLVATRNLLGAVKNDLSGLVAEQRGDDYAMSAGLE
RFLEKLYIAE-

S3 Text. Genomic DNA sequences of genes comprising the putative gene cluster for the biosynthesis of 1.

>FAD oxidoreductase
ATGGTTTCTCCAAAGAACTTCCTCTTTACGGTCGCTTCTACGGCATTGCTCGCCCATGCAGCCCC
TGCCGCTGATGGTGGAAGGTTGGCCGCAATCGCTGCTTGCGCGTTAGTCGGTGTGAGGCCCAG
CCTCGAGCCAGCCTTTGCTGGTGAGGAATCTGTTCAAAAGCCCTATAAACTTAGGCTAATACCTT
CATCTTTAGGCTCTTTACAGTACACCAACGACATCTCCCATTGGTCAAACGCTAGCAGTCTTCCTT
CGTTGTGTAGCGCTCAGCCAAATACGCCACAAGAAATCCAGACATTGGTACGCTATGCTACTATT
TTTACATGAAACGTGCTAATTCCTATCTTAAGTTCTCGATTATTAGGCAAACCCGTACCCCTTGGG
CAGTGAGTCCCCTCCTAATTGCCATGGTTTCATCTTTAATTCTTCCGCGTCCAGGTGAAAGGCAA
AGGTCACACCAACGTCAACAATGCGAGCTCTACATACGGTCTACAAATTGCCATGACCCAATTCA
ATCAGATCGAACTGAGCCCTGACAAGAAATCCGTAAAACTCGGCATGGGTCTCGATTGGGCGCA
GGTGTACACGGCCTTGGATCCGCACGGTGTCATCGTAGTTGGCGGCAGAGCTCCTGGAGTTGG
TATGTGGAAAGCATTACCTACGATTCGACTGAAAAACTAATCGAGTTCCTCAGGTGTGGCCGGAC
TGACCCTCAGCAGCGGATATGCCTGGTTCACCAACGAATATGGATTCGCGATCGACAACACTCT
CGCCGCTGGTACGCTTATTTTGACTAATCTCGAAGCTTATCAGAGCTTATCTGAATCGGTACCAG
ATATTGTTCTCCCTAACGGAACATTCACCACTGTCTCCGAGACACATGGTGCTGACATCTTCCGA
GGTCTGCAAGTAGGTGCCTTTTATTCGGCTGCTTCGCAGCATGCTTATGACGTTACAGGGCGGT
CTCAACAACTTCGGAATCGTCACTAGTATCACCTTGAAGACCTTCCCTCTTGGCCAAGTCTGGGC
TGGAGTCATGAACTATCCAGGCGAAGCCTTGGGGGCCTTGATCAACGTAACCGCTGAGTACTCT
GCGAAGCCTTCAAACCCCAAGGCTACTCTCGCCACCGAGTTCTTTTCCACTAACGGTTCTGTGA
GTACGATAAATCCGCCTTTTAATTTGATGCTAGAAGCGTTTACTCTATTTTTAGATGTCCGGTTTG
GCCGTTATCTTCTATGACGGCCCGACTCCCCCTCCTGGCCTCTTCGACGACTTCCTCACCATCC
CCGGTGCTAGCGGTACCGTCACCACAAGATCGTTCCTTGATTTCATCCTCACGAACGACGTTCC
CGGCGATCCCACCAACCGGTGTGTCTATATCCCCTGGCCCCTGTTCTCCTTATCTAATAGTAACA
CAGTGCTACCTGGTATTCTGCCCCTATCCTTACCTGGACTCGCGAGCTGCTCATTGCCTTGCAC
GACCTTTGCCGAGACTACTCCATCAAGGTGGCAAAGCTGAGCAGGAGTGGCACGCTCGTCTCA
GCCAATGTCGAACCTTTCGGTCCCCAGGCATACTCGTTCAACCGTGGCTCTGCATGGCCCCATG
ATCCCTCGTCTCCATACAACACCTTCAATGGTCTCACGATGTGGAGCGATCCTCGTGACGATAAG
CTCATGTTCAACCTCACCGTCGAATATCATACTGCTGTTTGGGACACTGCAGTCGCCCTTGGTAC
TTCCGACCGTGCAAGTACCGCGTACTTCCCCCCTAACTACGTGAGTAATAAAGATTAGGGGTCTT
CATCTGACTGATGTGCGTATAGGCTCAGAGTGTCACCCCTTCTCAGAGCATCTATGGCCCCAAC
ATGGGCTGGATGAAGGAGGTTAAGAGGAGGGTGGATCCTCATAACCTTATCAGCCTCACGGGC
GGCTACAAGATTTGAACCAACATTCTTTTACGTTCGATATCATTGCTGACGCTATTCCCCTCTCGT
TTCACAACATTCATTCTAATTACGCCGTCCTTATCTTCATCAGCTTTGCTTACTTATCTTTATTAAC
TTCACTTAACTCGCCCGTATTAATGCCATTCAACCCTGTTTACTTGTCATTGTACACTTCAAACCA
CAGTATGTATATACTACGGATAAAACAGTTTTACTGAACTTTCCATCTTTGTGAGAGCTACATTCA
CGTCCCCCGACCTCAAGAATGTCAAAGATATCTCTTTGGAAGTCAGAGCCAAGTCAGAGTGCTCT
CGAGAAGAGACACAAGAATGGAATGTCCTTTCGTCCGGGGCTTTCTCGCTTATGGTAAGTAATAC
CTTTGCTTCCAAAACCCTTCAGGGTTCTGGTGGAGCTCGGGATGGACGAACACCCGGTAAATCT
TTGATTAATGTCTGAGGCCAGTTCTAGTATTCATCGCTGGACACCTATTGATAGTGCTAAACGTAA
CTTCCTGAGAGTCTTTCAGACATTGGAAGCGAAGAACAGAGGATTAGGTCTTCGAAATCCTAGCA
TGTGAGCAGAAATACAAGAGTTCTCAGTATGGTACTGAGATCATTGAACACCGCGAACTTCTTGT
GAATGTCTCCGGGGATGGTAGCGCAGTCCAATGGCCTCCGGATGGTCCGTAGCATGATCTTAGA
GCTGAGCGATCCGAGCTTCCGGGTCTTATTTTCTCTTCTTTTTACGTGGCATAGTGAAACCTGAG
TGCCGAGAGTTAGTCTGGGATCTGGTGGGATCACCCTCCAATGATGGGTAACACATTTCTATTTG
GGGAACTACTGGCCCTCTAGATAGCTTGAGGATATGTCAACACAATGTCAACTTGGCCAGAGATT
CTTGTCCGACCACAAGGAAATCCTACGATCCTTCTTTGAAACTAAGATTGAGTCTTGAATCCGGA

CGATCTACTTTTGACGTTGAAAGGAAGACCTTGCTTCCCGCCTCACGCATGCGGGGTAGAAATC
ATTCGTTGAATGCGGCTGTGATTCGAGGAACGCGCGATGTCAAATGTCTTTGCGGAAGTTCCTC
AGGACCGATCCATGTGTGCCGCAAGAAGACTGGACACGCCCGTCTGATGATGCGAAGCTCGTC
ATCTACTAGGTAATAGTGTATCTCTTCTTATTTTTCCGGAAGTTCTTCCCGACTTCCGACACGATG
GTCAACCATGTCCGAGTCATCCGACGCCGGCGAATTCGAGGTGCGAACGGAGATTAAAGTTGCT
CAGAGATAACTTCGCATTAGCCGCGTCGAGTGCGAAGACTCGTCCATCATCATCTAACGCGGGT
TCGTGTACTTATTTTTGGCGAAGGATTGTCTTGGCATGCTCACAACAAGGGCGAGGGTCAATTTG
AAGTCTTTCTTTTTTTCCTTGCTGAGATGTGACCGTGGAATGTATGTATTGTCGAAGCAGGTATGT
GAATAATTTACGGGATCTAATATACTTCTCAATGAATGGTCTTCGAGCTTCAAGCTTCAAGTACAC
ACAGAGTCTCCACCGATAGGAAAAACGGAGAGATGGATTCTTAGTATAATCATTCTTTCCTTTATT
CTAAGTTCAGACGCGAAGTTGTAATCCATCCGTTATCGAAGTACGTTATGGGAATGAGATCTAGG
GCACTTTTGGATTTACTCAAGAGGATCGAGAGATATGGGATTTATGCCGCATTAGGAGGCAACG
CCACTGTCATTAGGAATGCGATTAAAACGTCCAAGGTAAGCTACTGGCGCCAATGGTTTCTCATC
GGCATCCGGAGTGTGTACCTTTACAAGATCTACTGGCGAACCACTGGCTACACCTCGAAACACC
CGGAGGGGACGGGGCTTGGCATCTGGTTGTCGGGGTCGATGTTCCCCATTCATGTAAAATTAGC
TTCATTGAGGAATGGCATAGGTTGTATCATAGTCATCGGAGGTGATAGGATGCAATGGTAGTCC

>sesquiterpene cyclase
ATGCCTGGATCAGCGGACTGGACTCCTGATAGATTCTACCTCCCGGACACATTGGCCAACTGGC
CTTGGCCCAGGGCAATCAACCCAGCATACGATGAATGCAAAGCAGCGTCGGCGGCCTGGTGTG
CCAAATATGGTGCCTTCTCAGCGCGTGCACAGAAAGCCTTCGACCTTTGCGACTTCAGTAAGTC
GTCCCTTAATTCGCCAACAAGAATTTACCGCTGACATGAAGTTATAATTCTCTAGACCTCCTCGCT
TCATTAGCCTACGCCCATCTCCCTGCTGGTGAGTTAAGATCCGTTCGCACTTAGATATCCGGGAC
TAATGCATCAAACACCACAGACGTCAACCGTGTGGGATGTGATCTTATGAACCTGTTTTTCGTGG
TGGACGAACACACCGATGCGATGGACGCGCGCTCCGTTCAGGAATGGGTGGATATTGTTGTTGA
TGCTCTCCATAATCCTCATAAGCCAAGGCCAGCAGGAGAGCCGATTGTTGGGGAAGTGGCGCG
CACGTATGCGGTCCTCATCATTGATTCCTTCAATGGAGATACTGATAGAAATCTTCGACTAGTTTT
TGGGAGAATGGCGTCAAGTGCCTGGGTCCCACCTCACGAAGTCGATTCGTTGAGACTTTTACTA
CCTACCTCCAAAGTGTCGTGACTCAAGCACAAGACCGAGACAATCACCTTTTCCGCGATGTGAA
CAGCTACATGGACGTGAGGCGAGACACCATCGGAGCCAAGCCGTCTTTTGCTCTCCTCGAGCAT
GACATGGAAATGCCGGATGAGGTATTCAATCATCCCCTCCTTGAGAACTTGAGAGAGTGGGCAA
TCGACATGCTTGTCATTGGAAATGTATGACCGGTCCATCCGCCATATTTTTTGTGATGAATTAACC
TCCTCTCCAGGACTTGTGCTCGTACAACGTCGAGCAATCTCGCAATGATGACGGTCACAATATCG
TCCGACTTGCCATGCTTCAAGAAAATACAAATATTCATGGCGCACTTCGTTTCGTCTCCAACATG
CACGATGATCTCGTGCAGAAGTTCCTTGACAACTACAAAAATATGCCTTCGTTCGGTCAGCTCAT
CGACGAATGGGTCTCCAGGTACATTGAAGGACTCGGCAATTGGGTCCGAGCCAATGACACATGG
AGCTTCGAGAGCTGGCGGTACTTCAAAGGTGATGGGTTGCGTGTCCAAGAGGAGCGCTGGGTA
GATCTGCTCCCCCCAGCTCCCAAGGACGAGCTCACTTCTTCCAGTACGTTATTCTTTACTCGACC
TTTATATCCGGGATTCTTACACTGTTGCTAGTTCCCCCAGAGTCGCGCTGGATCAAACCTGCCGT
CGAGCCAACCCGAGGAAGGCCCAATAATGTTGGTATTGTGGCGATGGACACATACTCTCCGACC
TCGGAGGAGCGCCTGGAGGGTGACTTCGAAACTTTGACTGTGAAAAGTAAGCCACCAGGATCC
GCCTTTCCCGGTTGTTGTCTCATAAACCCCAATTAGCCATTTCTTCTCTTCTATCAAAGTATAACA
TCAATCCCTCGTCCGTTGGGCGCCTGGATATCTGCATCGAGAAGTCTGCTGATCCCTACATCCT
CCCGGCTGCACGAGATGCCTTCGCCTCTGTCGGCAACACTGATGTCGAAGCCATTGTCAGCTCC
AACTCTGTCGTCGGACTCTTCAATTCGATCAACTGGGTTGAATCTTCCAGTTGGGATGGCCGCTT
TGCTATCGTATTTGGTGGAGATCTGACCTCCGGTACTTCGGCCGCTTTGGTCGGTCCTGATGCT
CCTATTGTCGTGGAACGTATGTCATTCACGCTCATTTCACCTTCTTCCGACAACTGACAACTCCTT
CCAGCGACCCGTGGAACCTACCTTGCAGATCCGTACGGATCGGCAGAAGAGGACACTGGCTCC
TACTTGGACTCCCTGTTCCAATCCTACTCCCACTACCGAAAGAAGCACCCACAGTTCTCCAAGGT
TCCAATTCTTGCGAGCCCCCTCCCCCGCGGCGTATCAAAACCTAAGGCACGCACCAACGGGACA
AACGGAAAAGGGCCTCGAACTCGTTCTTCTTCGACTACCTCCTCCACCGCTTCGAATAGTTCTGC
TCCCTCGTTCGACTACATGATCCTGCATGACAAGTACGGCCAGATCCCTACCGGAGCCGGATCA
ATTTACTCTGGCCTTGCCGGCCTGATTGCTGACCGGGACCCGGAGCAACTGCGAGGCAAGAAC
ATTGGTGTGTTCGGCTTTGGGAACAGTACCAGCACTTTCTTTGCAGTTCGTGTCGCTGGTGACTG
CAGCGTAATCCACAAGCGTCTCCGGTCACAACTTTAACTCAGATCTCCACGGATAGTCGTATTGC
GTAGGATGGGAGTAAGCCACAGCATATTGACTTCACGATTTACTATCATTACTACTAGATTTTTCG
AGCTCTCTCAATCATCCTTGTAACAGTTACATCCCCTAATTTTGGATTCATATGGCCGTACAGTAA
ACTCAATGCACTACTTCGAGCTGAGAACAATATTGATCTACCCGGTGTGATGTATTTCTGTTGCTT
TGAAGCAGCTAGTGGGAACCGATCCTAAGTTCATGTCGCGTTGAGGAGCAGAACTTGAATTTTG
AGGAGCTGTCAGTGGGGACACAACCCCATCATCCTACTTGGTTATCCCGATGAGCTGGAGTGCG

AGCATTCCCAGAAAGAGTCTGAAAGACAAATAACCTGCTTGTTTTTGGTAGGGCAGATTCACCGT
TTGTCAGCTGCCGTTACTACATACATAATACTTACGGCACCCGAGAGCTGTCTTTGAACAGTCTC
TCCGACTCGCCAAGTACGTGAGGGCCGGGGCACCAAACAGTGTCGTATCGTGCCCTCCCCCTT
GGAATACACTAGCCCACCGATCGTGGGAACATAATATTGTCACGTTGAGTGAGTTCGCAGGCCG
CTGTGACGAGAAACTTCGATTTCCATGAACATGAAGACTGGGTGCTCAAAAAAAAAAAGTCTCAT
ATCATACACAGGCCCGTGAAGGCACTCTAAATTTCGGAATTACAGTAACGCTACATGAAGTTTCC
ATGTATTACTGAATCAGAAACTTTCACACAGGGTATTCCTGTAGTGCAGGCCAATATCTGCCAATA
TGTGGGCTGGCTCCACTTTCTGCCTCAATCGTCCATGGACTCTGAGCGCGAGCTATCATGGACG
CGTCTGCTGATTGACACGGCTTTGGAAGCCTGGTATTCGACATCCGAAGGATCAATCCCTTGATT
CCTCAGCGAGCTGCGTATTCTGGACATGATATAGGATAAACATTCTCAATAGTTCTTTCAATCCAA
ATGGGACTCGCGAAATGGCGCCTTAAAGCCCCTATTTTCATCGAAATTACTGCTTCCGCGGTACG
CGCCTCGCAACCCCAAATGGAGACGTTTTCCTCTTAATAGGATACATGCAGCGCAAGCTTGCCG
GTCCTGTCAGATCTCTTCGACAGATTTTTGTATCTCATCCTCGAAGAGGTCGAAACAAAGTACGT
TTTTCGCCTAGGATACGACGCGTTAACAAATGCTGCCGACAAACGCGTGGGACCCTGA

>aldo-keto reductase
ATGACATTCTCATCCAGGATCCCTGTTATCTACGTGAGTTCCTAACCTTCGTTTATTTGAGTAGCT
ATTGATCTCTCTGCGATATCAAGGGCGCTGGTGGAATTGGTGCCCCAGGCACATTCTGCAAGCT
CACTAGCCCAGAGATTGCTCAACCCGTCATTGATGCCTGGTGCAAAATTACTGGTCCTTCTACGA
TCGACACCTCCAACCTCTACGGTTTCGGTTCTTCGGAGGTTATTCTTTCCCAAATGAACTTGCAC
GGATCCGTTATCGATACCAAGTACGGAGGTCCCTTGATCATAGATAACTCAGCCTCATTTCACTT
AATCCCAGGTGCTATCCCCTGGCCCCAGGCGACCACTCGTACGAGACGATCAAAGAGAAAGCC
AACGAATCCTTCGCCAAGCTTAAGGGCCTCAAGGTCCGCGTGTTCTACCTGCATGCTCCCGACC
GCAGCGTCCCTTTCAAGGAAACCCTCCGTGCCATTGACGAGCTTTATAAAGAAGGGAAATTGTAA
GCACTAACTTCATCGAAGTGACATACTCACTTATCGACACCCATGCCTGCTGCCTGCAGTGAGTA
CTTCGGTCTGAGCAACTACTACGCCTATGAAGTCGCCGAAATTGTGACCATCGCCCGTGAAAAC
AACTGGGTTGTCCCCACTGTGTACGAGGGTATTTACAACCCCATTGACCGTACGGCCGAAGTAG
AGTGCGTATAGCTCCTTGAATCATTACCCTCTTAGTCTGACAGGAGAGTTCCACAAATTTAGGTT
GATTCCAGCCCTCCGCCGCTACGGCATTCGCTTTGCTCCCTACTCAGTCTTGGCCGGTGGCCTC
CTTGTTGGCCATCTTCTCTCTGAGTCCGACGAACTTACTCAAGTCGAGGAAGGCAGCCACTACG
ATCCAAAACTCCCCTTCGGAACTTTTTTCAACACCCGTTACGGCGGTTTGATTCCTGAAGTCCGC
AAATTGAAGGAGAAGGTGGAGGCGGCAGGCCTTAACTTGAACCAGGCCTCTGTGAGGTGGTTG
CAGCACCACAGTGCCCTCTTGCCCAGTGACTTGGGTATCATTTTCGGAGGTAGCAAGCCCAGTC
ACGTTGAAAGAACTCTGCAATACAAGTGAGTAAGACGAACTATGAGAACTTGCAAGGAACTAACC
CACCATCAGTGCGGAGGGACCTCTTCCGGCCGAGGTTGTTGAGGCATTTGACGTCACCTACAAG
AATGTCAAAGCAACTCTCCCCAACTACAACCACAGCCCGCATTGGTATAATTTAAAGGAGTACGG
CTACTAA

>P450-dependent oxidoreductase
ATGAGCTCAATCTTCGAGAACCTGAGCAACCACCCCCTCGCCGTCTTGGGAGGTGCTGTTCTCC
TCATCGCCCTCCTTGCAAGCAAGCCAAAGAAGGGAAAACGACCTCCTGGTCCTCGTGGTCTGCC
ACTGGTATGTAAACTCTTATAATTCAGCACTAGTTCTCGGGGATTTACGTCGCATAGGTTGGCAA
TGTTTTCCAATTGTCTGAAGAGAATTGGATTACCTTCACCGAGTGGAAGTTCAAGTATGGTCCCA
TCGTCGGCCTGAACCTCGGTGGTAAGAACACCATCGTCCTCAACACCCACAAAGTAGCATCAGA
TCTCCTCGACAAGCGCGCATCTATCTATTCCGATAGTGAGTCGTTGCCTTCCTCTAAAGTCCTCC
CGTCTCATGCCAATAATTCACGATAGGACCTCGATTCATTGTCGCGCAGGAGATGCTTTGTGGTG
GTCTACTGCTCGTCTTCACCCGGTATGGTGATATGTACGTCCTGAAAGTTCATGCACTCCCTGCC
ATTTATTGACTTTTCGCTTACCCACAGGTGGCGTCGCATGCGTCGTGCTGCTCACGAAGGTCTCT
ACTCCCACGCTGCCGAGAGCTACTACACTCTCCAAGAAACTGAGGCAACTGTGCTCGTTGATGG
TATCCTCAAGTCGCCTGACGCTTGGGACGATCATCTGAAGCGTACTGCTGCATCCCAAATTATGG
CTATGGTTTACGATACCCTTCCCATCAAGGATCACAACGAACCCCTGATTAACCGTGTCAACGAC
CTCGTCCAACGTCTCGTCAAGGCAGCTTATCCCGGAGAGCACTTGGTCGAATTCTTCCCCTTCCT
TAACAAACTTCCTGATTGGATTGCCAAGTGGAAACCTTGGGCTGCCGAGTGGCACCGAAAGGAT
TCTGAACTCTTCATGGACTACTATAAGGCTGTCCGGGATCGTGTCGTGAGTTTCTGACGAATTCC
TCAAGTGAATATCCTTTCACACTTATTGCAGCTTGCTGGTGATGAACGTCCCAGTCTCGTGTCCT
CTCTTGTCAAGCGAAGTAAGGAGAACAAGTTCACCGAACGTGAGAAGTCGTGGGTTGCTGGAGT
CATGGTGTAAGTTTTCCAGCCTAACTTGTTTTAGCTGGCGCTTTACTCACCTGTCTCTCCGCCTTC
CAGTTCTGCAGGTGCCGAGACTACTGCTGCTGTGATGGCTTGGTTCTTCCTTGCTATGACCCTCT
ACCCTGAGGTCCAGCGCAAGGCACAGGAAGAGATTGACCGAGTCGTCGGCCATGATCGCATGC
CTAGCTTTGAAGACATCGACCAGCTGCCATACATGAGGGCCATGGTCCGCGAGATCTTGCGATG

GCGCCCGGTAGATCCTATCGGTCTCCAACATCAGTCTACGGAAGACGACGTCTACGAGGGTTAC
TTCATCCCCAAGGGATCACTCGTTATCTTCAACGTCTGGTGTGTATTAGCTCAAGTTTGCACCAT
CTGGCTATAATTGATCGTTCTCTTAGGGCAATGAACCGTGACCCCGAAATCTACGGCGAAGACTT
TGAGGAATTCAAACCCGAGCGATTCCTTGACGAAGATGGTCAACTCAAGCCCACTCATCCCGCT
ACCAAAGGAGAAGGCCACGTAACTTACGGCTTCGGTCGTCGTATCTGTGTCGGTCGCTATGTCG
CCAACAACACCCTCTTCATTGATATGGCCCATGTCCTTTGGGCTTGCTCGATTGAAAAGGCAAAG
AACCCTGACGGGACCGATATCAACTATGACTCCTATGCCAACCACAACGAGGGTCTCGTCGTGT
ACGTTGTTCTTTCTCTTGACTTCGATTTAGGATACTGATTATTCTCAATTCGTGTAGTCGTCCTGC
CTACTTCAAGAATGTTATCAAGCCCCGGTTCCCCGCGGCGGCCCAGATAGTTGCAGAGCAGAAG
GAGCGCCTCATTTCTGCTGCCAGCGAAGGCAAAACAATCAGAATTTCTGGAACTTCATTCCAGGA
TGTTTAA

S1 Table. $_{13}$C and $_{1}$H NMR data of bovistol B (1) in d$_4$- MeOH.

position	δC (ppm)*	δH-ppm (J, Hz)	Key HMBC
1	150.0		
2	119.1		
3	133.3		
4	124.5		
5	142.1		
6	43.1	2.83/2.52(d/d, 15.8, 15.8)	C5, C7, C9, C10, C11,
7	45.0		
8	40.1	2.70/2.55 (d/d, 16.2, 16.2)	C1, C5, C7, C9, C10, C11,
9	127.4		
10	70.7	3.40(s)	C11, C7, C6, C8
11	24.9	1.16(s)	C10, C7, C6, C8
12	15.0	2.13(s)	C3, C4, C5
13	32.8	2.80(t, 8.3)	C2, C3, C4, C14
14	61.8	3.50(m)	C13, C3
15	20.6	2.77/2.36 (ddt, 16.1, 4.6)	C2, C1, C3, C15’, C2’
1’	198.9		
2’	80.6		
3’	31.6		
4’	144.9		
5’	158.5		
6’	43.8	2.94/2.39(d/d, 17.8, 17.8)	C5’, C7’, C9’, C10’, C11’,C1’
7’	43.7		
8’	40.7	2.57/2.39 (d/d, 17.0, 17.0)	C5’, C7’, C9’, C10’, C11’,C1’
9’	135.1		
10’	70.4	3.41(s)	C11’, C7’, C6’, C8’
11’	24.9	1.14(s)	C10’, C7’, C6’, C8’
12’	113.3	5.29/5.25 (s/s)	C4’, C3’, C5’
13’/14’	12.9	1.38/0.26 (ddd, 16.7, 6.5.5.2)	C3’, C4’, C2’, C14’/C13’
13’/14’	5.31	1.13/0.80(ddd, 16.7, 6.5, 5.2)	C3’, C4’, C2’, C13’/C14’
15’	30.0	2.19/1.87 (ddt, 16.1, 4.6,)	C1’, C15, C2, C2’, C3’

S2 Table. ^{13}C and ^{1}H NMR data of D (2) in d_4- MeOH.

position	δC (ppm)*	δH-ppm (J, Hz)	Key HMBC
1	150.2		
2	119.0		
3	133.8		
4	124.4		
5	141.3		
6	44.8	3.36/2.75(d/d, 15.1, 15.1)	C5, C7, C9, C10, C11,
7	45.1		
8	41.6	3.23/2.76 (d/d, 15.2, 15.2)	C1, C5, C7, C9, C10, C11,
9	126.6		
10	182.2		
11	26.3	1.37(s)	C10, C7, C6, C8
12	15.3	2.14(s)	C3, C4, C5
13	32.8	2.81(OL[1])	C2, C3, C4, C14
14	61.5	3.50(m)	C13, C3
15	20.5	2.78/2.36 (ddt, 4.6, 16.1)	C2, C1, C3, C15’, C2’
1’	198.9		
2’	80.8		
3’	31.8		
4’	144.4		
5’	158.5		
6’	43.7	2.94/2.39(d/d, 17.8, 17.8)	C5’, C7’, C9’, C10’, C11’,C1’
7’	43.8		
8’	40.5	2.56/2.39 (d/d, 17.0, 17.0)	C5’, C7’, C9’, C10’, C11’,C1’
9’	135.1		
10’	70.3	3.42(s)	C11’, C7’, C6’, C8’
11’	24.8	1.15(s)	C10’, C7’, C6’, C8’
12’	113.4	5.29/5.25 (s/s)	C4’, C3’, C5’
13’/14’	13.7	1.31/0.25 (ddd, 16.7, 6.5.5.2)	C3’, C4’, C2’, C14’
13’/14’	5.4	1.12/0.80(ddd, 16.7, 6.5, 5.2)	C3’, C4’, C2’, C13’
15’	29.9	2.19/1.88 (ddt, 4.6, 16.1)	C1’, C15, C2, C2’, C3’

S3 Table. ^{13}C and ^{1}H NMR data of strossmayerin (7) in d_4- MeOH.

position	δC (ppm)*	δH-ppm (J, Hz)	Key HMBC
1	147.4		
2	120.9		
3	131.3		
4	123.8		
5	141.4		
6	42.8	2.80/2.51(d/d, 15.5, 15.5)	C4, C5, C7, C8, C9, C10, C11,
7	45.1		
8	40.1	2.78/2.51(d/d, 15.5, 15.5)	C1, C5, C6, C7, C9, C10, C11,
9	125.6		
10	70.4	3.42(s)	C6, C7, C8, C11
11	24.6	1.14(s)	C6, C7, C8, C10
12	14.2	2.02(s)	C3, C4, C5
13	27.1	2.63(t, 6.0)	C2, C3, C4, C14
14	65.9	3.90(t, 6.0)	C13, C3, C15
15	65.6	4.70 (s)	C1, C2, C3, C13, C14

[1] Over-lapping signal

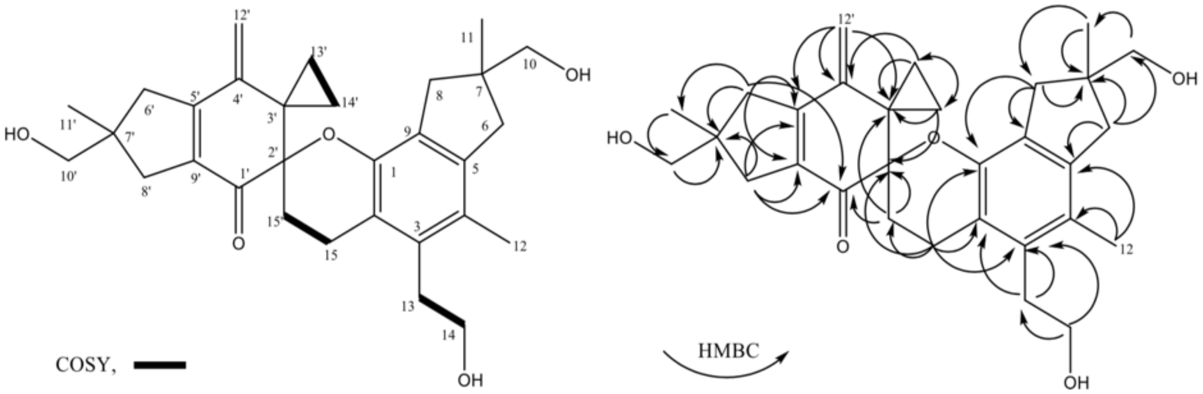

S1 Figure. Key 2D NMR correlations of bovistol B (1) showing COSY HMBC in d_4- MeOH.

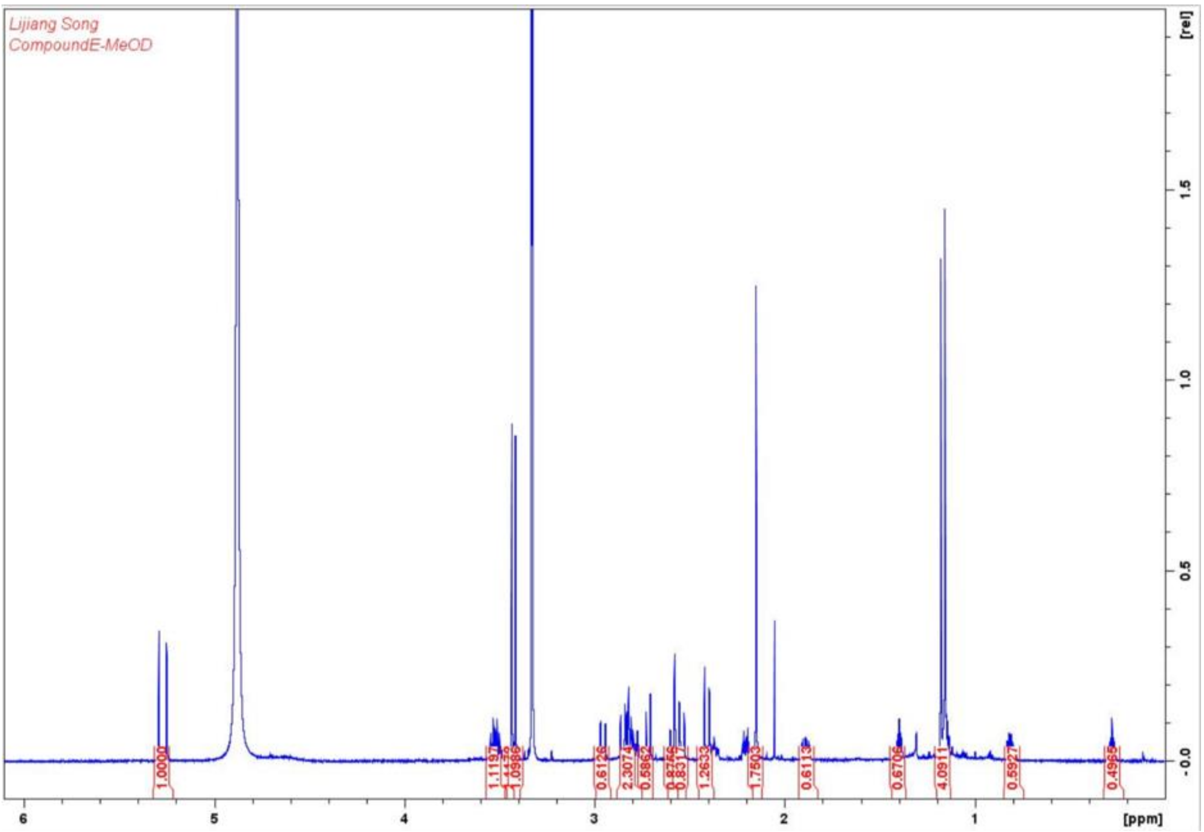

S2 Figure. ^{1}H NMR spectrum of bovistol B (1) in d_4- MeOH.

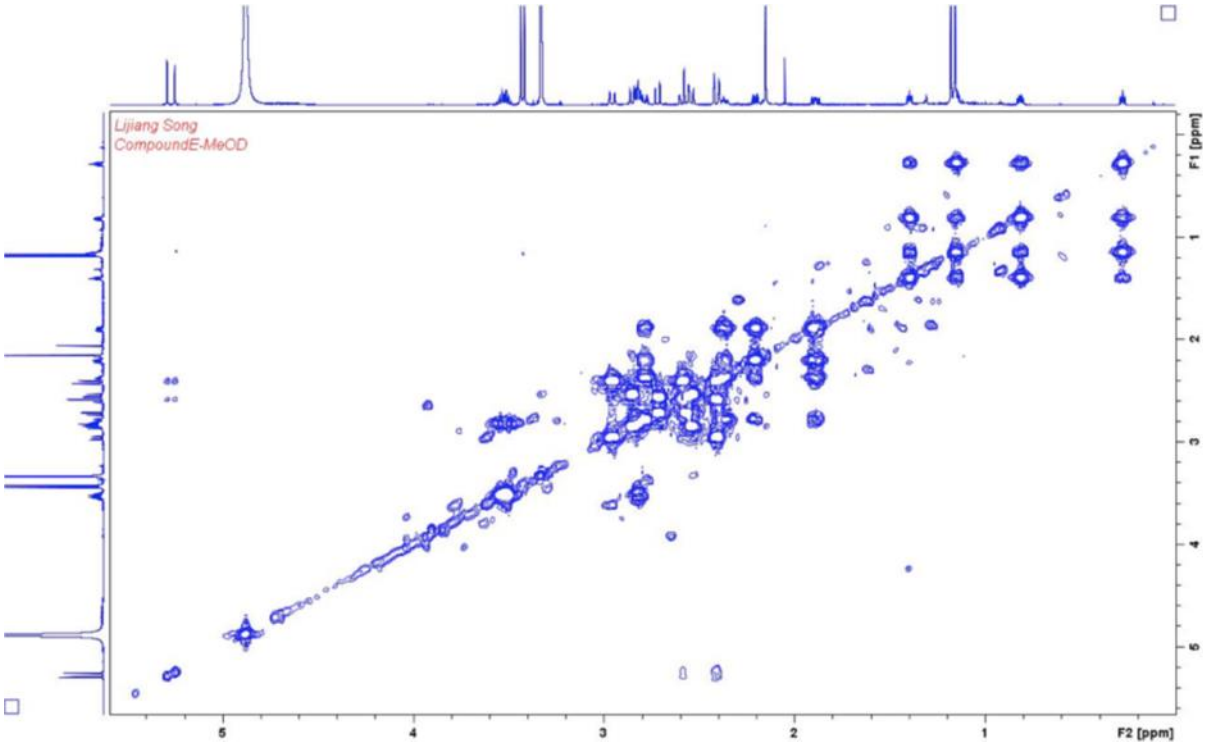

S3 Figure. COSY NMR spectrum of bovistol B (1) in d4- MeOH.

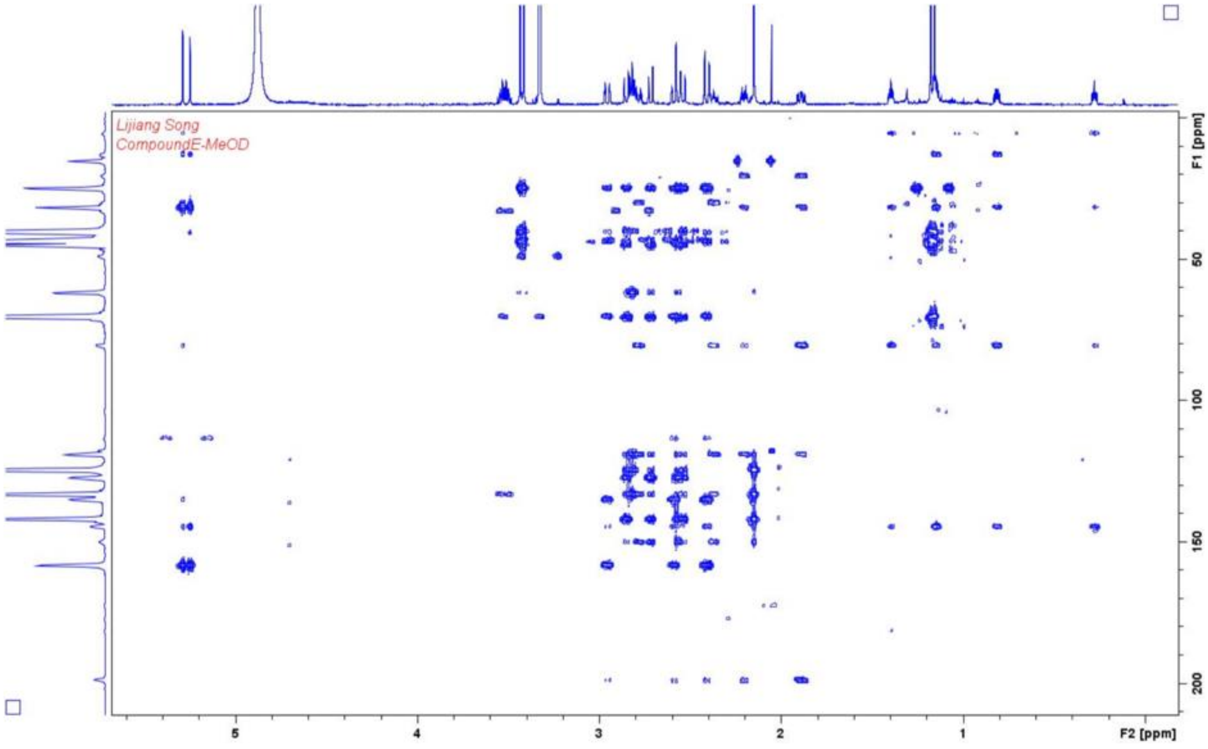

S4 Figure. HMBC NMR spectrum of bovistol B (1) in d4- MeOH.

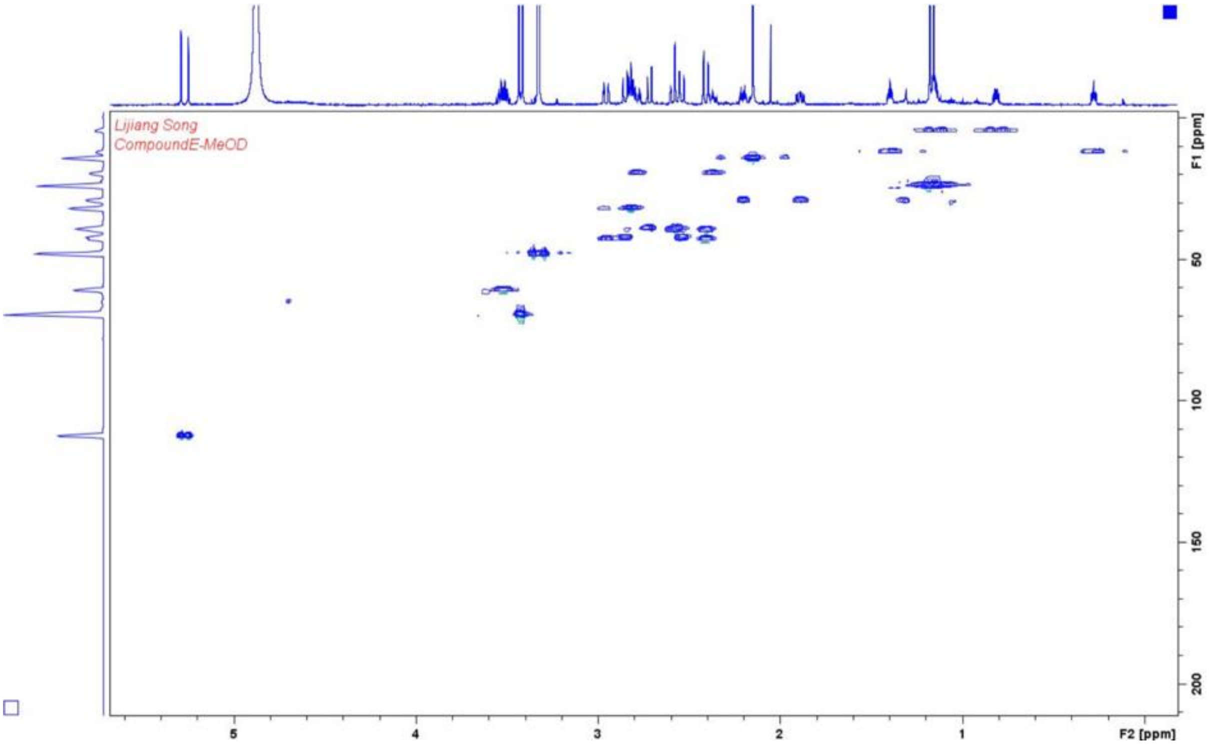

S5 Figure. HSQC NMR spectrum of bovistol B (1) in d4- MeOH.

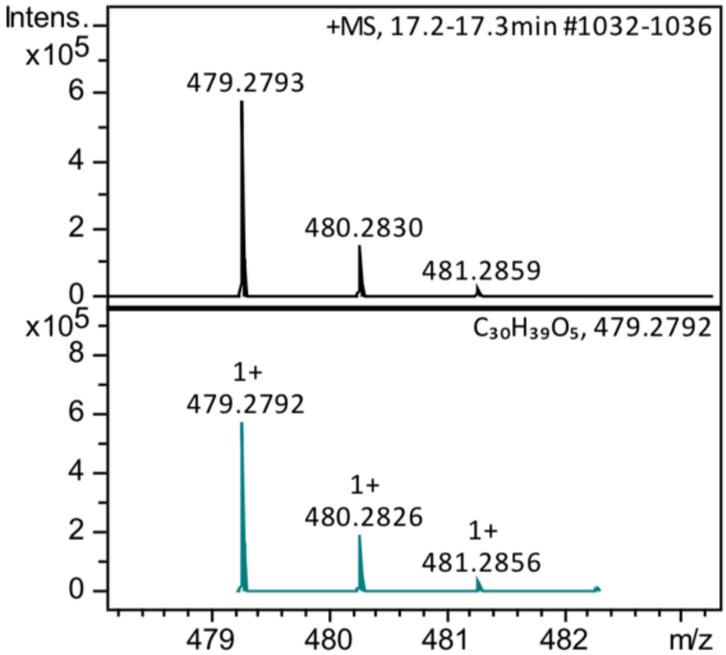

S6 Figure. HRMS of bovistol B (1). Top panel: measured spectrum, bottom pannel: simulated spectrum.

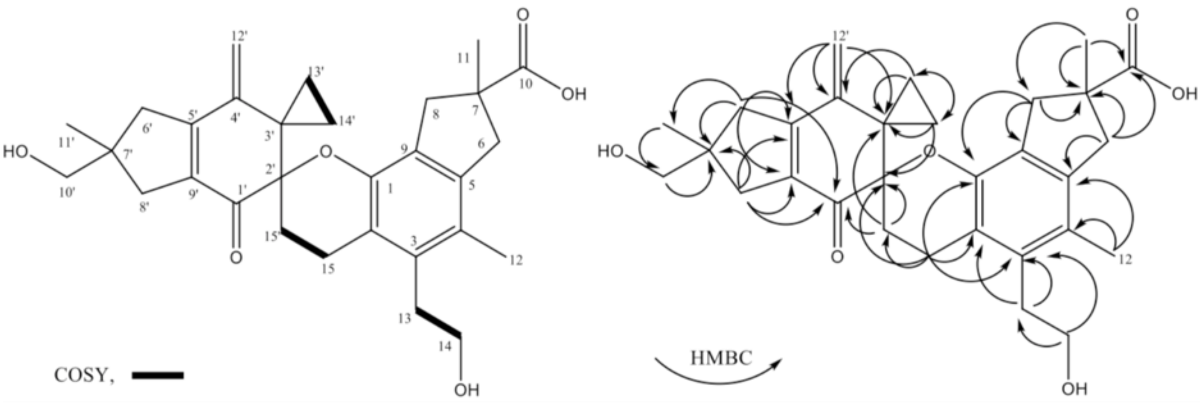

S7 Figure. Key 2D NMR correlations of D (2) showing COSY HMBC in d_4- MeOH.

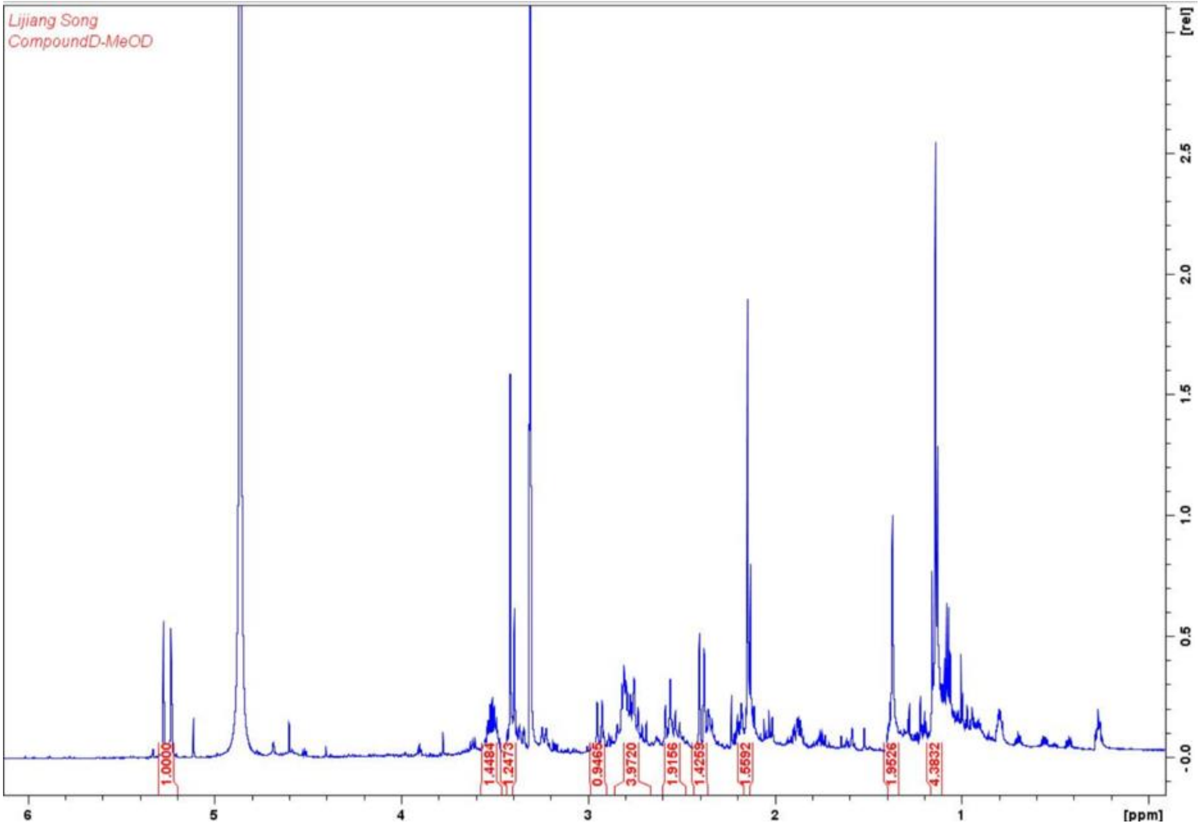

S8 Figure. ^{1}H NMR spectrum of D (2) in d_4- MeOH.

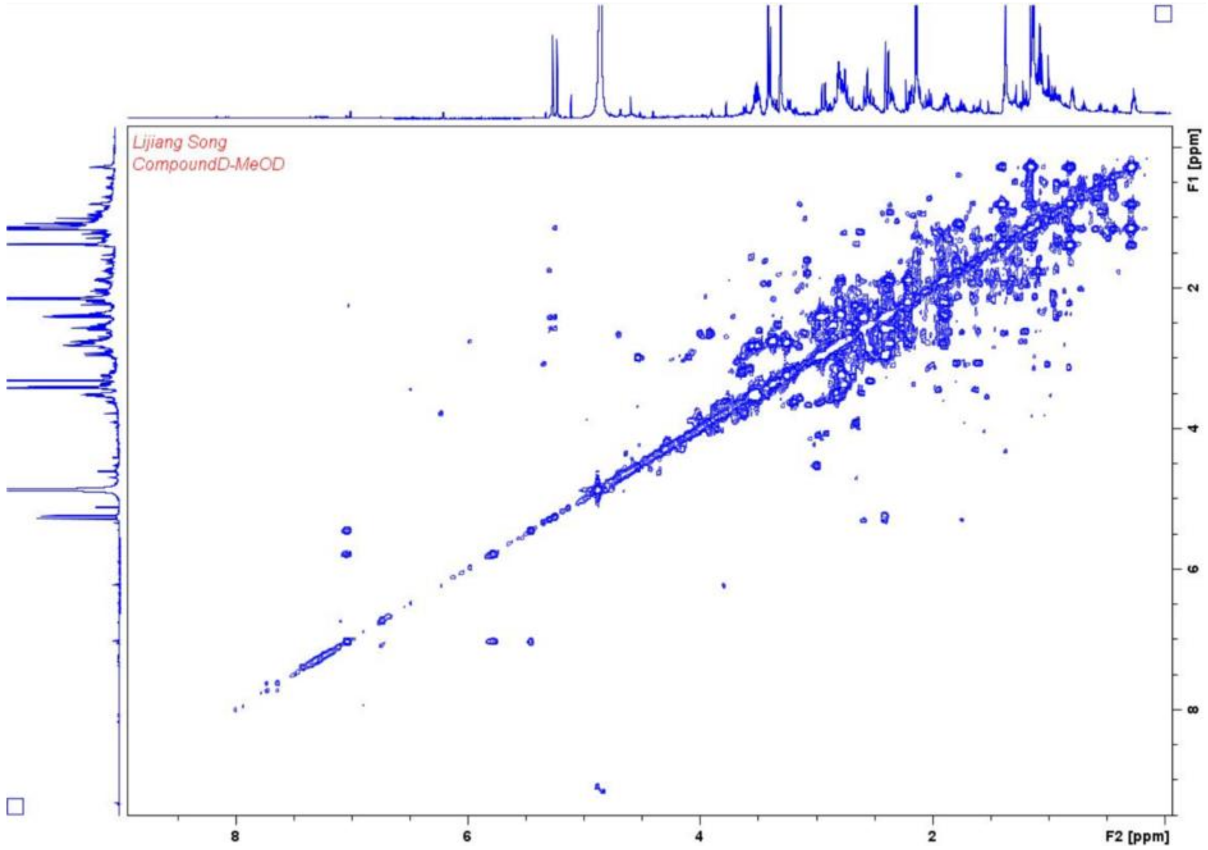

S9 Figure. COSY NMR spectrum of D (2) in d4- MeOH.

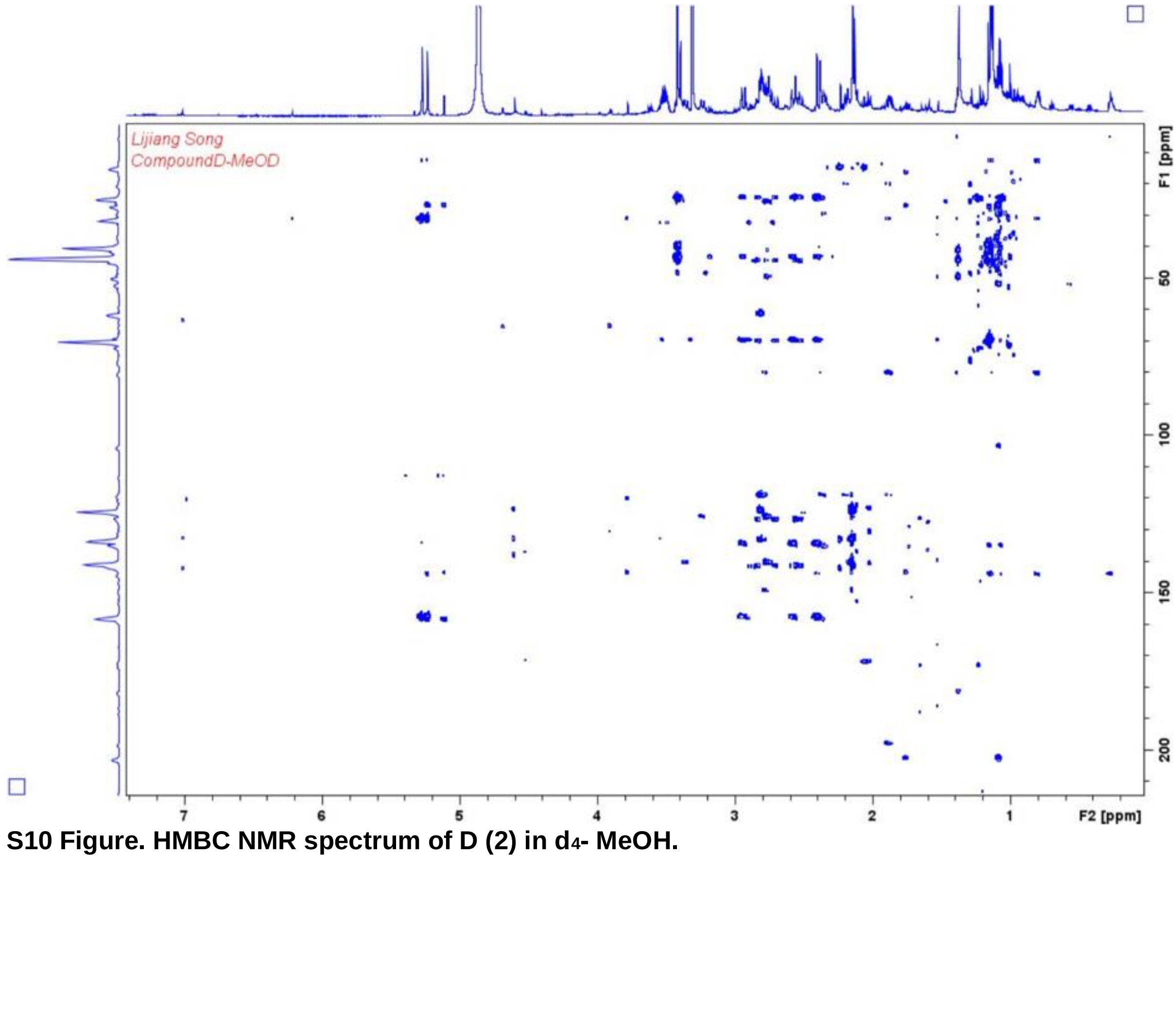

S10 Figure. HMBC NMR spectrum of D (2) in d4- MeOH.

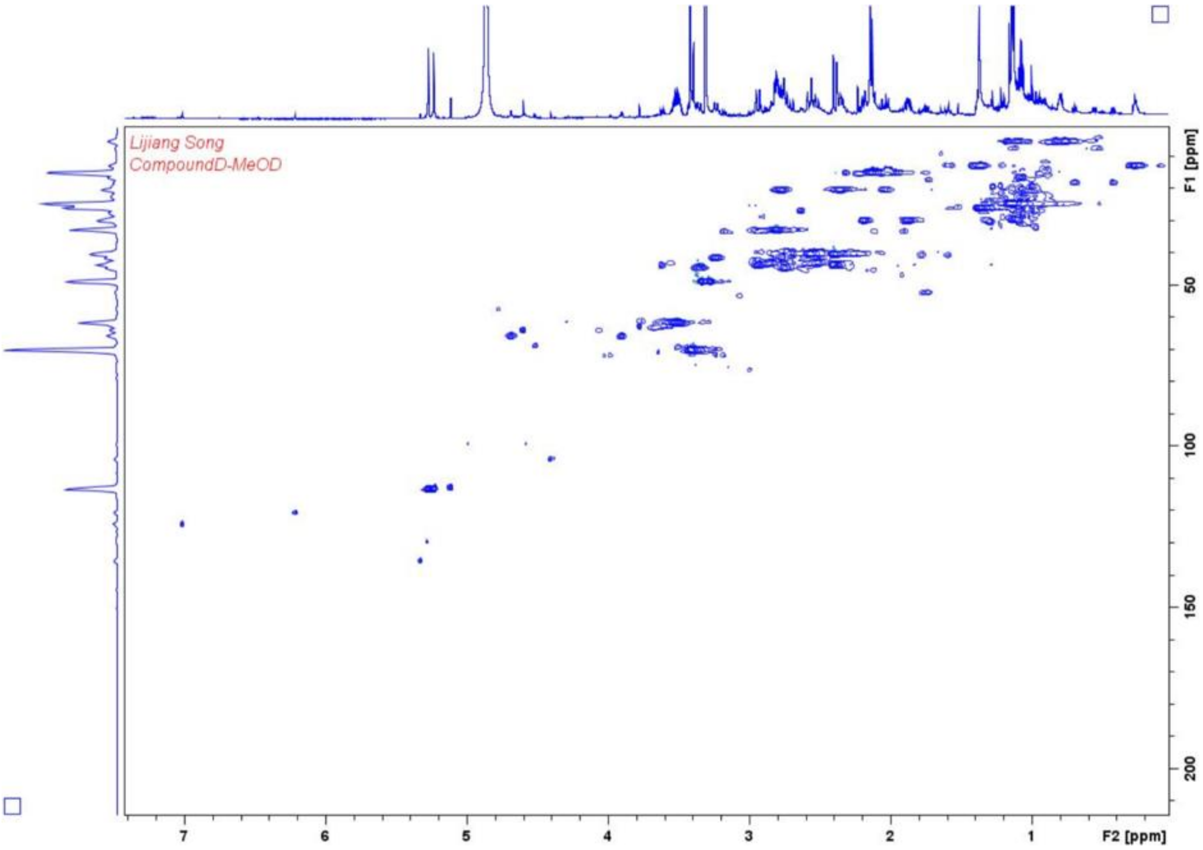

S11 Figure. HSQC NMR spectrum of D (2) in d4- MeOH.

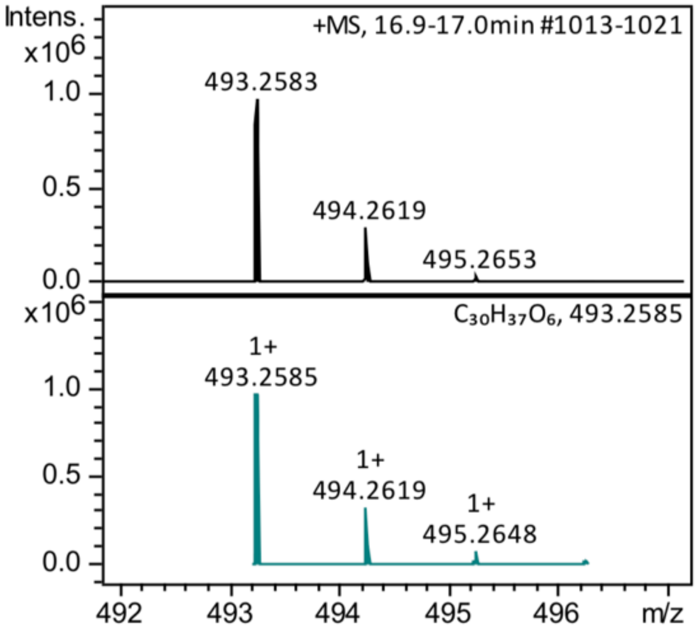

S12 Figure. HRMS of D (2). Top panel: measured spectrum, bottom pannel: simulated spectrum.

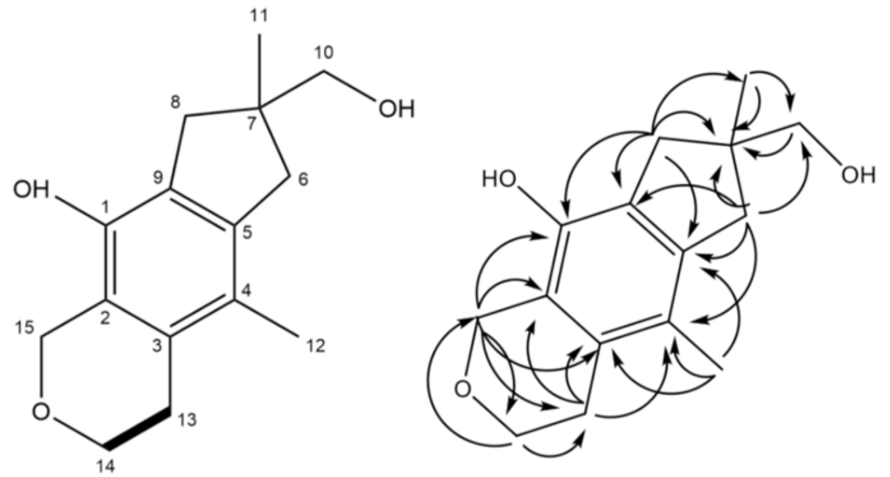

S13 Figure. Key 2D NMR correlations of strossmayerin (7) showing COSY HMBC in d_4- MeOH.

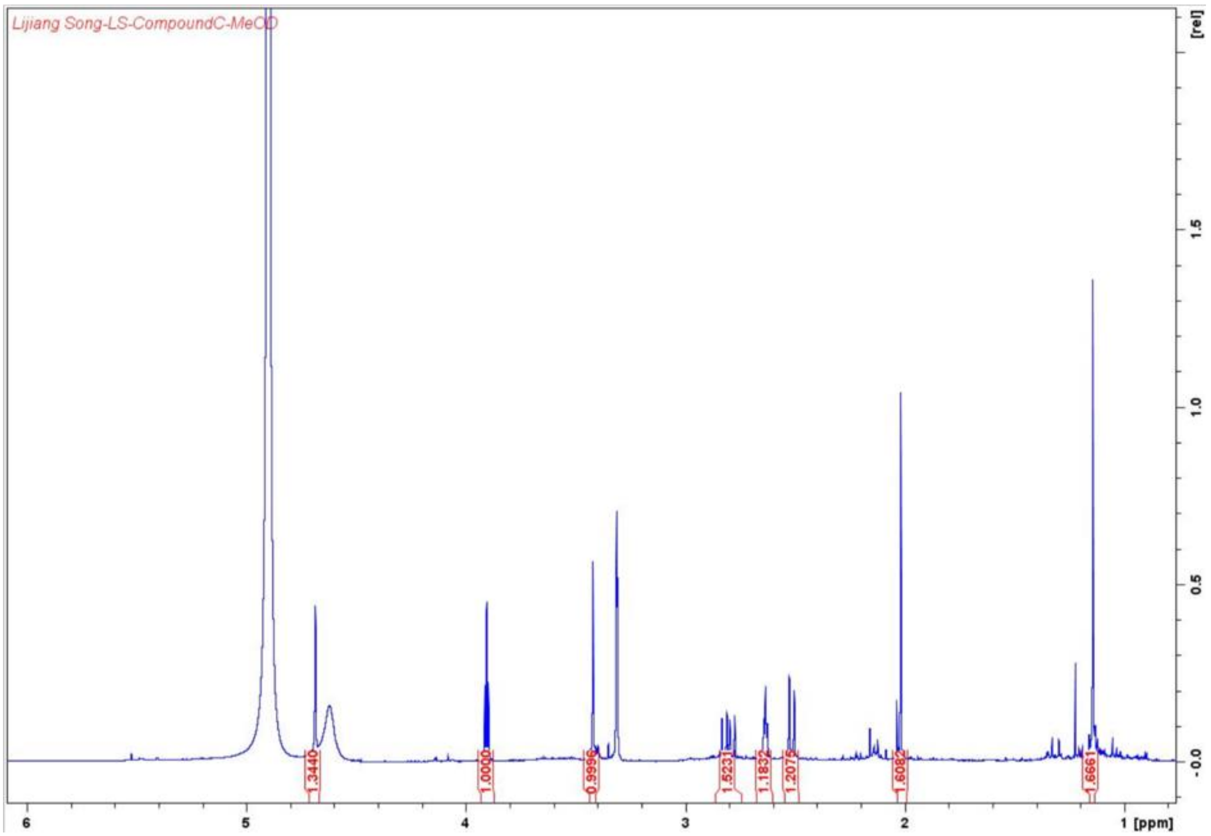

S14 Figure. ^{1}H NMR spectrum of strossmayerin (7) in d_4- MeOH.

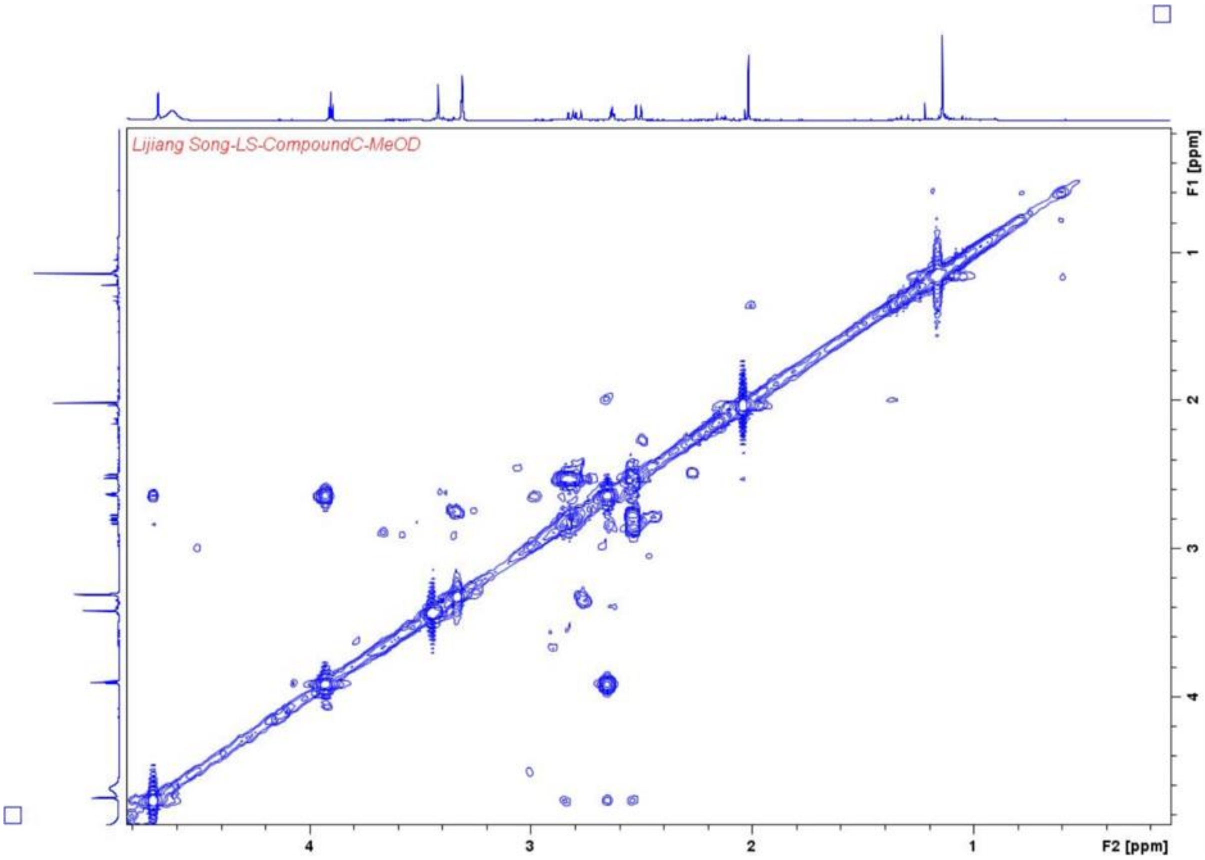

S15 Figure. COSY NMR spectrum of strossmayerin (7) in d4- MeOH.

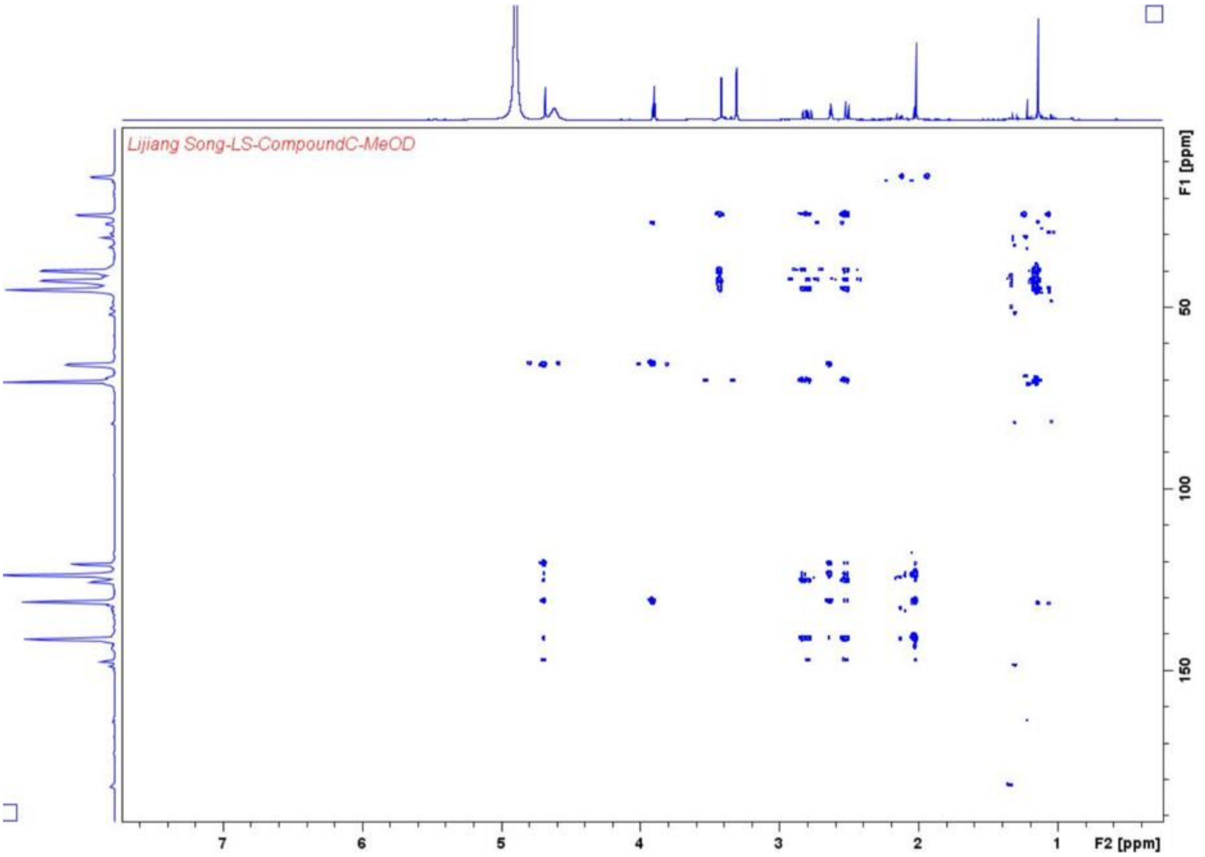

S16 Figure. HMBC NMR spectrum of strossmayerin (7) in d4- MeOH.

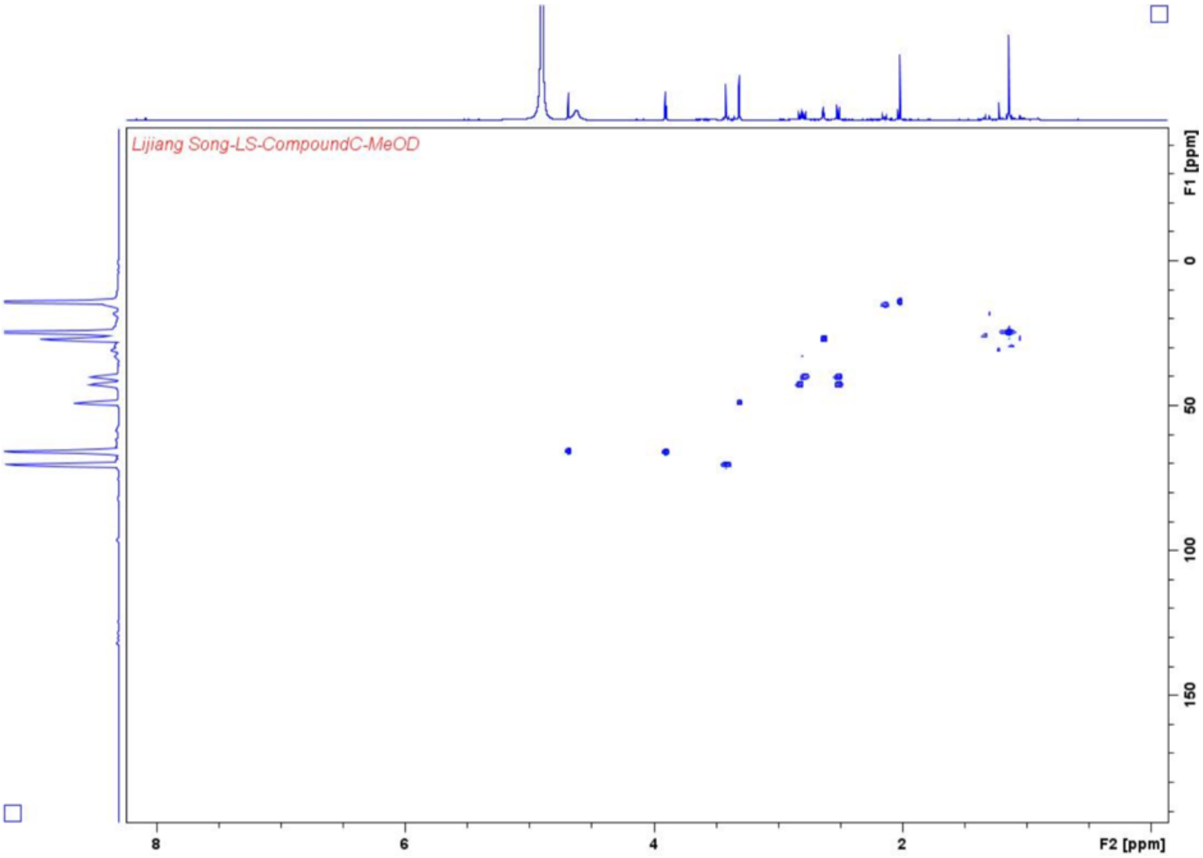

S17 Figure. HSQC NMR spectrum of strossmayerin (7) in d4- MeOH.

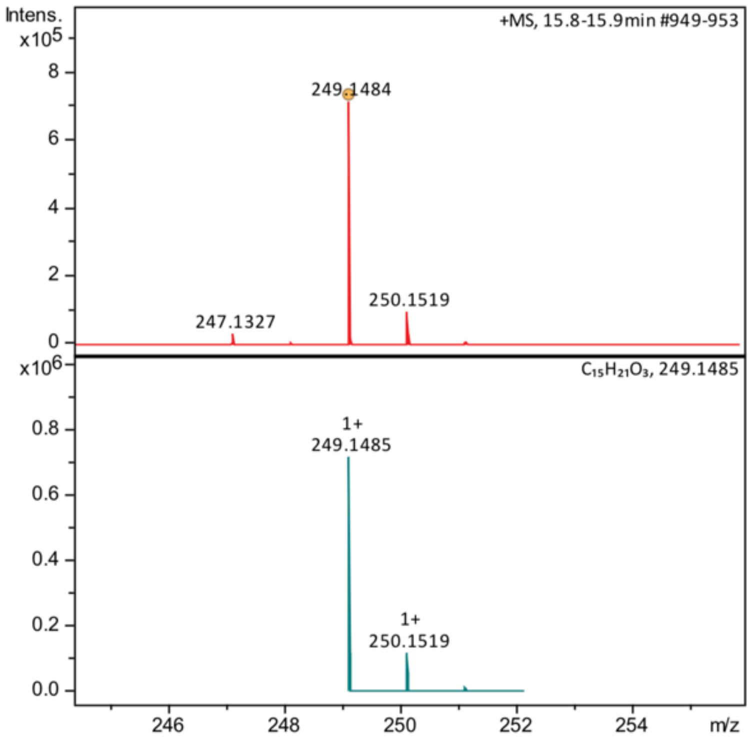

S18 Figure. HRMS of D (2). Top panel: measured spectrum, bottom pannel: simulated spectrum.

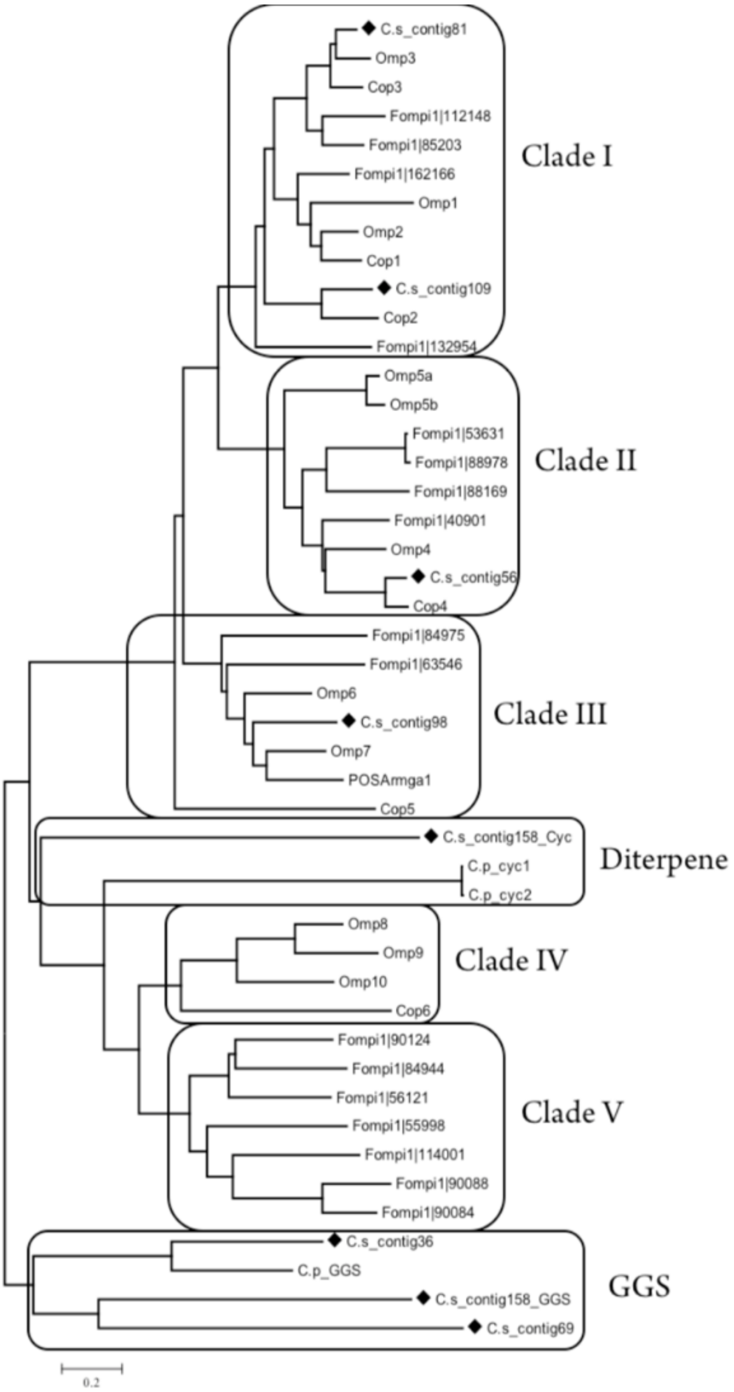

S19 Figure. Phylogenetic analysis of terpenoid cyclase homologues from *Coprinopsis strossmayeri* (C.s). Genes from *C. strossmayeri* have been compared to characterised sesquiterpene synthases from *Coprinopsis cinerea* (Cop), *Omphalotus olearius* (Omp), *Armillaria gallica* (Armga), and *Fomitopsis pinicola* (Fompi1) [32], and the GGS and diterpene cyclase sequences from *Clitopilus passeckerianus* (C.p). Sesquiterpene clades are indicated in the unrooted neighbor-joining phylogram and accession numbers, contigs, or gene references are indicated aside species abbreviations. The evolutionary history was inferred using the Neighbor-joining method [20]. The optimal tree with the sum of branch length = 20.43 is shown. The tree is drawn to scale, with branch lengths in the same units as those of the evolutionary distances used to infer the phylogenetic tree. The evolutionary distances were computed using the Poisson correction method and are in the units of the number of amino acid substitutions per site [33]. The analysis involved 46 amino acid sequences. All positions containing gaps and missing data were eliminated. There was a total of 210 positions in the final dataset. Evolutionary analyses were conducted in MEGA 5.0 [21].

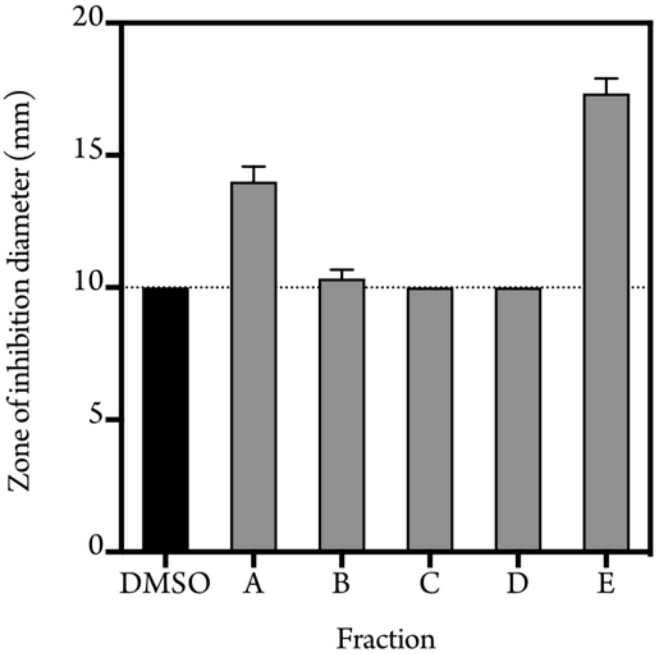

S20 Figure. Antimicrobial activity of fractions A-E against *Bacillus subtilis*. Fractions A-E were tested at 1 mg/mL for antimicrobial activity against *B. subtilis* ATCC 6633, resulting zones of inhibition were compared to the DMSO solvent control. *P* values were generated by ANOVA using the Dunnett's Test for multiple comparisons to one control. Data show the mean ± SEM (n = 3). This revealed significant inhibition in fractions A and E (both $P < 0.0001$).
